# Successful reinforcement history suppresses explicit and implicit error corrections

**DOI:** 10.1371/journal.pcbi.1014574

**Published:** 2026-08-03

**Authors:** John Buggeln, Nicholas Muscara, Seth R. Sullivan, Jan A. Calalo, Truc T. Ngo, Matthew Short, Adam M. Roth, Michael J. Carter, Joshua G. A. Cashaback

**Affiliations:** 1 Department of Biomedical Engineering, University of Delaware, Newark, Delaware, United States of America; 2 Biomechanics and Movement Science Program, University of Delaware, Newark, Delaware, United States of America; 3 Department of Mechanical Engineering, University of Delaware, Newark, Delaware, United States of America; 4 Department of Kinesiology, McMaster University, Hamilton, Ontario, Canada; Pantheon-Sorbonne University: Universite Paris 1 Pantheon-Sorbonne, FRANCE

## Abstract

A skilled basketball player expects to make many shots. After a miss, they could make a larger correction because the miss was “surprising.” Alternatively, they could make only a small correction since their average past performance was successful. Despite its relevance, past work has not elucidated whether or how a history of reinforcement influences explicit (cognitive) and implicit (involuntary) error corrections. Across three reaching experiments, we compared two competing hypotheses: 1) reward prediction errors (“surprise”) modulate explicit and implicit error corrections, or 2) expected value (average reward) modulates explicit and implicit error corrections. Our experimental results and computational modelling support the idea that the expected value of a long-history of reinforcement modulates explicit error corrections. Further, we find that an immediate-history of reinforcement directly modulates implicit error corrections, which could not be explained by an independent task error model. Collectively, we find that a successful reinforcement history suppresses explicit and implicit error corrections.

## Author summary

Conflicting previous work has suggested that reinforcement feedback leads to either greater or smaller movement corrections. Here we elucidate that past successful actions lead to a suppression of future cognitive and involuntary movement corrections. Highlighting how multiple learning processes interact can lay the groundwork for more informed and effective neurorehabilitation.

## Introduction

After missing the strike zone, an ace baseball pitcher adjusts the next pitch depending on their accuracy and the current number of strikes or balls. Considering their prior successes and failures (reinforcement history) informs their corrections to motor errors (distance from the plate). Despite knowledge that key reinforcement computations depend on history [1,2], it is unclear how reinforcement history specifically interacts with error corrections. Understanding the interactions and temporal dynamics of sensorimotor learning processes is not just a theoretical exercise, but can generate neurorehabilitation improvements [3–5].

Error corrections are widely thought to consist of an explicit (cognitive) and an implicit (involuntary) process [6–12]. The explicit process is driven by performance errors, the difference between desired and actual performance. Performance errors lead to changes in explicit action selection that can improve performance. The implicit process is driven by sensory prediction errors, the difference between the sensory prediction and the sensory consequence of a movement [13–17]. Sensory prediction errors update a representation of the dynamics [13,14,18–22], which recalibrates the sensorimotor system and reduces motor errors.

Reinforcement contributes to human motor learning alongside error corrections. When given rewarding binary feedback in response to task success, participants adjust motor behaviour to maximize rewards [17,23–28]. Reinforcement feedback independently regulates movement variability [29–32], recalibrates the sensorimotor system, [33] and drives plastic changes in the motor cortex [34,35]. Two critical quantities are tracked by the reinforcement system: expected value and reward prediction error [2,36–41]. Expected value is the representation of the expected average reward. Reward prediction errors are phasic dopaminergic signals that encode the difference between expected value and reward valence (magnitude of received reward). Colloquially, reward prediction errors encode reward “surprise”.

Past work examining the interaction of error corrections and reinforcement feedback has conflicting results. A greater reward frequency has been shown to interfere with error corrections [42]. Confusingly, it has also been shown that greater reward increases error corrections [43]. This inconsistency could be a consequence of differences in reinforcement feedback type, variations in the involvement of explicit and implicit error corrections, and/or influence of reinforcement history. How reinforcement history and the associated computational quantities affect explicit and/or implicit error corrections is unclear.

One possibility is that expected value modulates error corrections, which would optimize average success. Previous work has suggested that we select actions that result in greater expected reward [44]. Another possibility is that reward prediction errors modulate error corrections, which would minimize surprise. Past work in rodents has shown that reward prediction errors regulate movement variability during reinforcement tasks [45].

Here, we tested whether reinforcement history modulates error corrections through expected value or reward prediction errors. In **Experiment 1** we manipulated reinforcement history to test whether expected value or reward prediction errors modulates explicit and/or implicit error corrections. Our empirical results and computational modelling support that expected value modulates error corrections. Yet, Experiment 1 does not parse whether that effect is through explicit and/or implicit processes. In **Experiment 2**, we isolated implicit error corrections and found no influence of a long-history (all previous trials) or short-history (a few previous trials) of reinforcement. Collectively, Experiment 1 and 2 suggest that expected value modulates only explicit error corrections. Prior work suggests reinforcement may modulate implicit error corrections, but these studies did not provide or manipulate extrinsic reinforcement feedback [46,47]. Therefore, in **Experiment 3** we further tested whether an immediate-history (previous trial only) of reinforcement or punishment influenced implicit error corrections. Empirical data and computational modelling support that the immediate-history of reinforcement modulates implicit error corrections directly through reward valence, the signed reward magnitude. Taken together, our results suggest that expected value modulates explicit error corrections, and an immediate-history reward valence modulates implicit error corrections.

## Results

### EXPERIMENT 1

The goal of Experiment 1 (N = 40) was to manipulate the reinforcement history to test whether expected value or reward prediction errors modulates explicit and/or implicit error corrections. To this end, we manipulated both the long-history of reinforcement and short-history of reinforcement.

#### Experiment 1 design.

**Fig 1:**
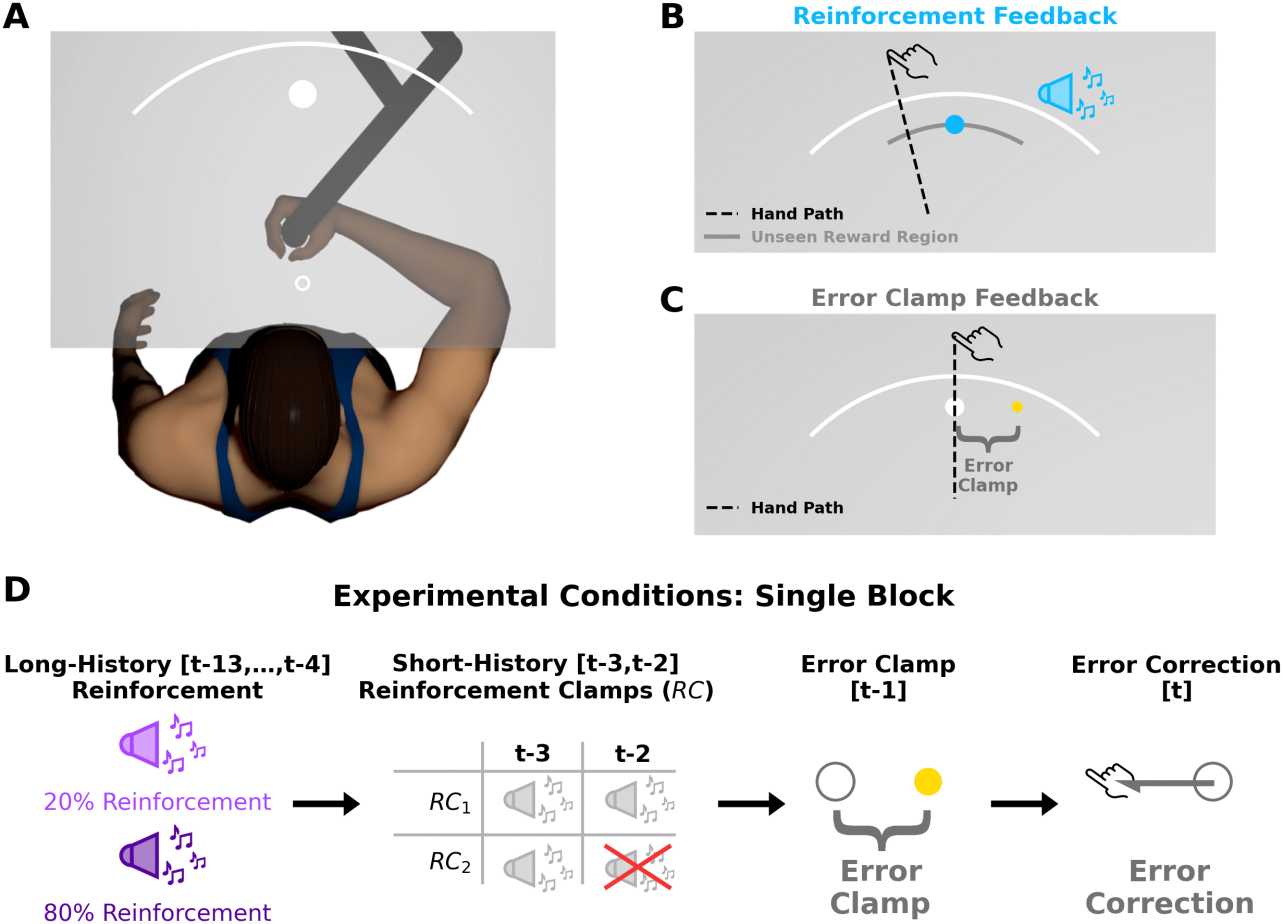
Experiment 1 & 2 Design. **A)** In all experiments, participants held the end of a robotic manipulandum and made reaching movements in the horizontal plane with no vision of their hand. Participants reached from a home position (open white circle) and quickly passed through a target (solid white circle) and boundary (thin white arc). The boundary arc disappeared once crossed and indicated a sufficient reach extent. **B)** In Experiment 1 & 2, participants were informed if they hit the target they would receive reinforcement feedback (target turned blue, expanded, a pleasant noise played, monetary bonus). They were also told that reinforcement feedback would be withheld if they missed the target. Unbeknownst to participants, the reinforcement feedback was probabilistic and was not contingent on hitting the visual target. **C)** During error clamp trials a cursor was shown as participants passed the target. Unbeknownst to participants, the cursor position was an experimentally imposed error in a fixed location independent of their hand position. **D)** Reinforcement history was manipulated within 48 blocks of trials. Shown above is a single block of trials. To manipulate the long-history of reinforcement, two groups of participants received either a 20% or 80% probability of reinforcement when their hand passed through an unseen reward region (thin grey arc). This probabilistic reinforcement feedback was provided from the 13th to 4th trial prior an error correction (i.e., [t-13,...,t-4]). If their hand failed to intersect the unseen reward region (thin gray arc) they would receive no reinforcement feedback. To manipulate the short-history of reinforcement, all participants experienced reinforcement clamps (*RC*) on the 3rd and 2nd trial prior to an error correction (i.e., [t-3,t-2]). The first clamp (*RC*_1_) provided two successive trials with reinforcement feedback. The second clamp (*RC*_2_) provided reinforcement feedback and then no reinforcement feedback. No endpoint feedback was given on short-history reinforcement clamps. Following the short-history reinforcement clamps, participants were shown an error clamp [t-1]. An error clamp was either within, left of, or right of the target. We measured error corrections to the left and right error clamps on the next trial **[t]**. In Experiment 1 participants were instructed to “hit the target,” which would include both explicit and implicit error corrections. In Experiment 2, participants were instructed to maintain a constant explicit strategy (“always aim to the target center and ignore the cursor feedback”) to isolate implicit error corrections.

In all experiments participants saw a circular home location, a single circular target, a thin boundary arc, and had no vision of their hand (Fig 1A). Participants were instructed to “hit the target” by reaching quickly through the target and boundary arc then stop.

The long-history of reinforcement feedback was manipulated by providing probabilistic reinforcement feedback in the first ten trials [i.e., t-13,...,t-4] of repeated experimental blocks (Fig 1D). Participants’ reinforcement probability was determined by a group randomization into either a 20% or 80% probability of reinforcement group. Participants received reinforcement probabilistically if they reached through a hidden reward region (Fig 1B) [30,31]. They were naive to the probabilistic nature of the feedback. Participants were told if they hit the target they would receive reinforcement feedback (target changed color and expanded, a pleasant sound played, participant received monetary reward).

The short-history of reinforcement was manipulated by providing all participants reinforcement clamps (Fig 1D). These clamps fixed the reinforcement feedback the two trials [i.e., t-3, t-2] before an error clamp [i.e., t-1]. Specifically, participants either i) received reinforcement feedback on both trials, or ii) or reinforcement feedback on the first trial and no reinforcement feedback on the second trial.

The last trial [t-1] of every block was an error clamp (Fig 1C). When given an error clamp, participants were briefly shown cursor feedback of their hand position when they passed by the target [48]. Unbeknownst to the participants, this endpoint cursor feedback was experimentally controlled to be a set distance from the target center. We experimentally controlled the error size to assess participants’ error corrections on the next trial [i.e., t]. The error corrections in response to the error clamps were our main dependent measure. If reinforcement history influences error corrections, these corrections should differ between the 20% and 80% probability of reinforcement groups and between the short-history reinforcement clamp types.

#### Experiment 1 models - *a priori* predictions.

We made *a priori* model predictions of error corrections. A first-order approximation of the error correction process is: [13,14,20,24,49,50]


$$\begin{array}{ccc} e_{t} & = & \left(T-X_{t}\right) \end{array}$$ (1)

$$\begin{array}{ccc} X_{t+1} & = & X_{t}+\beta e_{t} \end{array}$$ (2)

where $e_{t}$ is the error signal between the motor target, *T*, and the sensory feedback of the executed movement $X_{t}$. β is the learning rate on the error signal. $X_{t+1}$ represents the adjustment taken by the motor system.

Next, we considered that the reinforcement system may modulate error corrections through expected value (*EV*) or reward prediction error (*RPE*) as described by the following:


$$\begin{array}{ccc} EV_{t+1} & = & EV_{t}+\gamma RPE_{t} \end{array}$$ (3)

$$\begin{array}{ccc} RPE_{t} & = & \alpha r_{t}-EV_{t} \end{array}$$ (4)

*EV* for the motor target is updated by the $RPE_{t}$, multiplied by a learning rate (γ) at the end of each trial. The $RPE_{t}$ is the difference between the received reward and expected reward ($EV_{t}$). The received reward is the reward valence (α), the signed reward magnitude, multiplied by the presence of reward ($r_{t}$). We consider how $RPE_{t}$ or $EV_{t+1}$ can modulate error corrections in the following hypotheses.

Expected Value Model: One hypothesis is that expected value directly modulates the error correction term in Eq. 2, as follows:


$$\begin{array}{c} X_{t+1}=X_{t}+\left(1-EV_{t+1}\right)\beta e_{t} \end{array}$$ (5)

If past performance is unsuccessful (e.g., 20% reinforcement probability group), $EV_{t+1}$ is low. The expected value hypothesis predicts a low *EV* leads to large error corrections (Fig 3A). In other words, error corrections are “encouraged” because the expected value of the current action is low. Conversely, when past performance is successful (e.g., 80% reinforcement probability group) $EV_{t+1}$ is high. Therefore, error corrections are “discouraged” because the expected value of the current action is high. Under this hypothesis, *a priori* predictions (β = .65, γ = .01, α = 1) show error corrections are smaller if past performance is successful and larger if past performance is unsuccessful.

Reward Prediction Error Model: Alternatively, another hypothesis is that Reward Prediction Error modulates error corrections:


$$\begin{array}{c} X_{t+1}=X_{t}+\left(1-RPE_{t}\right)\beta e_{t} \end{array}$$ (6)

When past performance is unsuccessful (e.g., 20% reinforcement probability group), $RPE_{t}$ is a small negative number after a miss. The reward prediction error hypothesis predicts a small negative *RPE* leads to small error corrections. In other words, when past performance is unsuccessful, a miss is “unsurprising” so there is a small error correction (Fig 3A). Conversely, when past performance is successful (e.g., 80% reinforcement probability group) $RPE_{t}$ is a large negative number after a miss. The reward prediction error hypothesis predicts a large negative *RPE* leads to big error corrections. Therefore, when past performance is successful, a miss is “surprising” so there is a big error correction. Under this hypothesis, *a priori* predictions (β = .3, γ = .01, α = 1) show error corrections are smaller if past performance is unsuccessful, and larger if past performance is successful (Fig 3B). Critically, the Reward Prediction Error Model has opposing predictions to the Expected Value model with respect to reinforcement history.

No Modulation Model: Finally, there may be no modulation of error corrections by either reinforcement learning quantity (Fig 3C). This simply recovers the single rate state space equation (Equation 2).

#### Experiment 1 results.

**Fig 2:**
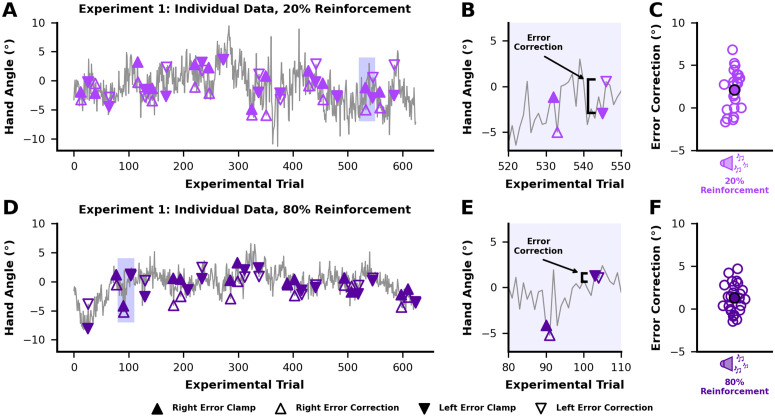
Experiment 1 Representative Individual Data. Hand angles (y-axis) over experimental trials (x-axis) for representative participants from both the **A)** 20% reinforcement group and **D)** 80% probability of reinforcement group. Error clamps that were pseudorandomly interleaved are denoted by solid triangles, and the subsequent error correction is denoted with an open triangle. To better illustrate how an error clamp leads to an error correction, the data shown within the purple shaded rectangles in **(A)** and **(D)** are expanded in **(B)** and **(E)**, respectively. All error corrections (y-axis) for the **(C)** 20% reinforcement participant and **(F**) 80% reinforcement participant. Note, these data show both leftward and rightward error corrections, where all rightward error corrections are multiplied by -1. As a result, both leftward and rightward error corrections are represented with positive values. Here, the 20% reinforcement participant had greater error corrections than the 80% reinforcement participant.

In Experiment 1 we manipulated the reinforcement history to test whether expected value or reward prediction errors modulate explicit and/or implicit error corrections. Fig 2 shows the hand angle over time of two separate participants in the 20% and 80% probability of reinforcement groups. Participants responded to left and right error clamps by making error corrections. In response to the same size error clamps, the participant in the 20% Probability of Reinforcement group made larger error corrections than the participant in the 80% Probability of Reinforcement group.

**Fig 3:**
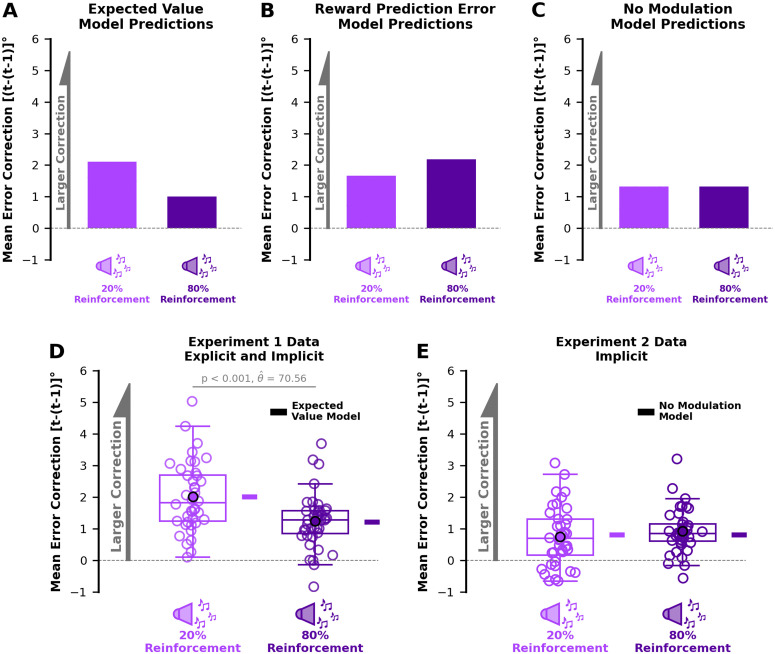
Experiment 1 & 2 Predictions and Results. **A-C)** *A priori* model predictions for the **(A)** Expected Value Model, **(B)** Reward Prediction Error Model, and **(C)** No Modulation Model for Experiment 1 and 2. Plotted are the mean error corrections (y-axis) after imposed error clamps for the 20% reinforcement and 80% reinforcement groups (x-axis). Mean error corrections are collapsed across short-history clamps, as there was no main effect of short-history. **A)** The Expected Value Model predicted that the 20% reinforcement group would have greater error corrections than the 80% reinforcement group. **B)** Conversely, the Reward Prediction Error Model predicted that the 20% reinforcement group would have smaller error corrections than the 80% reinforcement group. **C)** The No Modulation Model predicts no difference between the 20% reinforcement and 80% reinforcement groups. **D)** In Experiment 1 and aligned with the Expected Value Model, the 20% reinforcement group had greater error corrections compared to the 80% reinforcement group. Here, the shown behaviour is a composite of explicit and implicit error corrections. The best-fit Expected Value Model is denoted with small rectangles. **E)** To isolate implicit error corrections in Experiment 2, participants were instructed to maintain a constant explicit strategy, i.e., “always aim to the target center and ignore the cursor feedback”. No difference was found between the 20% and 80% reinforcement groups, which was best explained by the No Modulation model. Hollow circles are individual data points. Box and whisker plots are drawn for group data. Solid circles are the group-level averages. Collectively, these data from Experiment 1 & 2 support the idea that a long-history of reinforcement modulates explicit error corrections based on expected value, but does not modulate implicit error corrections.

We found a main effect of long-history of reinforcement (F(1,38) = 7.35, p = 0.010, $\eta^{2}$ = 0.16). There was no main effect of a short-history of reinforcement (F(1,38) = 3.51, p = 0.069, $\eta^{2}$ = 0.08), nor an interaction between long-history or short-history of reinforcement (F(1,38) = 0.073, p = 0.78, $\eta^{2}$ = 0.08). We confirmed that participants in the 20% and 80% Reinforcement group had different actual reward rates (18.35% vs. 67.60%, respectively, p < 0.001). We found that the 20% Reinforcement group had significantly larger error corrections than the 80% Reinforcement group (p = 0.001, $\hat{\theta }=70.56$, Fig 3D). The best-fit Expected Value model was able to capture our experimental results (Fig 3D), unlike the Reward Prediction Error Model or the No Modulation Model. Collectively, our empirical results and computational modelling support that expected value modulates error corrections.

Given that expected value is a temporally evolving quantity, we examined how error corrections changed across the course of the entire experiment in **Supplementary C** in the S1 Appendix. We found evidence that expected value’s modulatory effect on error corrections reached steady state within the first 8 error clamps.

### EXPERIMENT 2

In the first experiment, we instructed participants to “hit the target.” As a result, the sensorimotor system may have used explicit and/or implicit error corrections. In Experiment 2 we sought to isolate the influence of reinforcement processes on *implicit* error corrections. Thus, the goal of Experiment 2 (N = 40) was to manipulate the reinforcement history to test whether expected value or reward prediction errors modulate implicit error corrections.

#### Experiment 2 methods.

To isolate implicit error corrections, we repeated Experiment 1 with one crucial difference to the task instructions. Participants were instructed to maintain a constant explicit strategy, “always aim to the target center and ignore the cursor feedback” [48].

#### Experiment 2 models - *a priori* predictions.

It is well-established that error-based learning is composed of an explicit and an implicit process [8]. If the implicit process is isolated, we can use the same models as described above specifically for the implicit system.

Expected Value Model:


$$\begin{array}{c} X_{t+1}^{implicit}=X_{t}^{implicit}+\left(1-EV_{t+1}\right)\beta^{implicit}(T-X_{t}^{implicit}) \end{array}$$ (7)

Reward Prediction Error Model:


$$\begin{array}{c} X_{t+1}^{implicit}=X_{t}^{implicit}+\left(1-RPE_{t}\right)\beta^{implicit}(T-X_{t}^{implicit}) \end{array}$$ (8)

No Modulation Model:


$$\begin{array}{c} X_{t+1}^{implicit}=X_{t}^{implicit}+\beta^{implicit}(T-X_{t}^{implicit}) \end{array}$$ (9)

These models make the same qualitative predictions that were explicit/implicit agnostic but specifically for implicit corrections (as shown in Fig 3).

#### Experiment 2 results.

In Experiment 2 we manipulated the reinforcement history to test whether expected value or reward prediction errors modulate *implicit* error corrections. Unlike the first experiment, we found no main effect of the long-history reinforcement (F(1,38) = 0.32, p = 0.439, $\eta^{2}$= 0.02). Similar to the first experiment, we found no influence of short-history of reinforcement (F(1,38) = 0.29, p = 0.59, $\eta^{2}$ = 0.01) or an an interaction between the short-history and long-history (F(1,38) = 0.13, p = 0.73, $\eta^{2}$ = 0.00). We again confirmed that participants in the 20% and 80% reinforcement group had different actual reward rates (19.11% vs. 68.70%, respectively, p < 0.001). Thus, the results in Experiment 2 do not support that a long-history or short-history of reinforcement modulate implicit error corrections (Fig 3D).

Next we tested whether the Expected Value, Reward Prediction Error, and No Modulation models could best explain the data. As noted above, we did not see that a long-history or short-history of reinforcement influenced error corrections. However, a null result does not disprove a hypothesis. Best-fit parameters of all three models could be tuned to obtain no difference between experimental conditions. Indeed, all three models captured the results, while producing similar mean squared error for Experiment 2 (Table 1). Therefore, the best model was selected through a combination of mean-squared error and penalizing over-parameterization, using the Akaike Information Criterion (AIC) and Bayesian Information Criterion (BIC). A lower AIC and BIC indicate better models. The No Modulation model outperformed the Expected Value Model and Reward Prediction Error on both AIC and BIC (Table 1). Our behavioural and computational results support the No Modulation model. In the No Modulation model, a long-history or short-history of reinforcement does not influence implicit error corrections.

**Table 1:** AIC and BIC Analysis for models across all three experiments. Models were evaluated based on multiple criteria. Models were first evaluated on their ability to capture statistically significant experimental trends. Models capable of capturing trends were then compared using the Akaike Information Criterion (AIC) and the Bayesian Information Criterion (BIC). AIC and BIC both score models based on Mean Squared Error (MSE), while punishing for the number of parameters. Smaller scores indicate relatively better models. Rows shaded in grey indicate the best-fit model of each Experiment. All model fits are shown in Supplementary B in the S1 Appendix.

			Captures			
Experiment	Model	Parameters	Experimental Result	MSE	AIC Score	BIC Score
1	Expected Value	3	✓	1.82	30.11	35.19
1	Reward Prediction Error	3	✕	—	—	—
1	No Modulation	1	✕	—	—	—
2	Expected Value	3	✓	1.22	13.84	18.19
2	Reward Prediction Error	3	✓	1.20	13.32	18.38
2	No Modulation	1	✓	1.22	9.96	11.65
3	Reward Valence Floored	2	✓	0.84	-1.15	1.65
3	Expected Value Floored	3	✓	0.84	0.87	5.07
3	Reward Prediction Error Floored	3	✓	0.84	0.87	5.07
3	Task Error	2	✕	—	—	—
3	No Modulation	1	✕	—	—	—

Synthesizing the first two experimental results provides insight into the influence of reinforcement history on explicit and implicit error corrections. Experiment 1 showed that expected value modulated error corrections, but it was unclear to what degree explicit *and/or* implicit error corrections were contributing to the differences between the 20% and 80% conditions. In Experiment 2, we isolated implicit error corrections and found no modulation by either a long-history or short-history of reinforcement. Error corrections are composed of an explicit correction, an implicit correction, and any explicit-implicit interaction. In Experiment 2, we eliminated explicit corrections and therefore isolated implicit error corrections from either the explicit correction and any explicit-implicit interaction. We did not find any direct influence of the long-history or short-history of reinforcement on implicit corrections. Critically, this means the influence of the long-history of reinforcement in Experiment 1 must be a consequence of explicit error corrections.

### EXPERIMENT 3

In Experiment 2, we found no influence of the long-history or short-history of reinforcement on implicit error corrections. However, this does not rule out the influence of an immediate-history. In Experiment 2, we did not provide reinforcement on the same trial as a miss error clamp (i.e., on the [t-1] trial). In Experiment 3, we provide reinforcement on the same trial as a miss error clamp to investigate the immediate-history of reinforcement. We also chose to investigate the immediate-history of punishment. Importantly, previous literature shows inconsistent effects of reinforcing stimuli given on the same trial as a visual error [46,47,51], which we address with a relatively larger sample size and within subjects design. In short, the goal of Experiment 3 (N = 30) was to test whether the immediate-history of reinforcement or punishment influences implicit error corrections.

#### Experiment 3 methods.

Participants had identical instructions to Experiment 2 (“always aim to the target center and ignore the cursor feedback”) to isolate implicit error corrections. The immediate-history of reinforcement was manipulated by providing reinforcement on the same trial as error clamps [i.e., t-1]. Likewise, the immediate-history of punishment was manipulated by providing punishment on the same trial as error clamps [i.e., t-1]. When given punishment feedback, participants were told if they missed the target it would turn red, they would hear a negative buzzer sound, and they would lose money from a monetary bonus.

**Fig 4:**
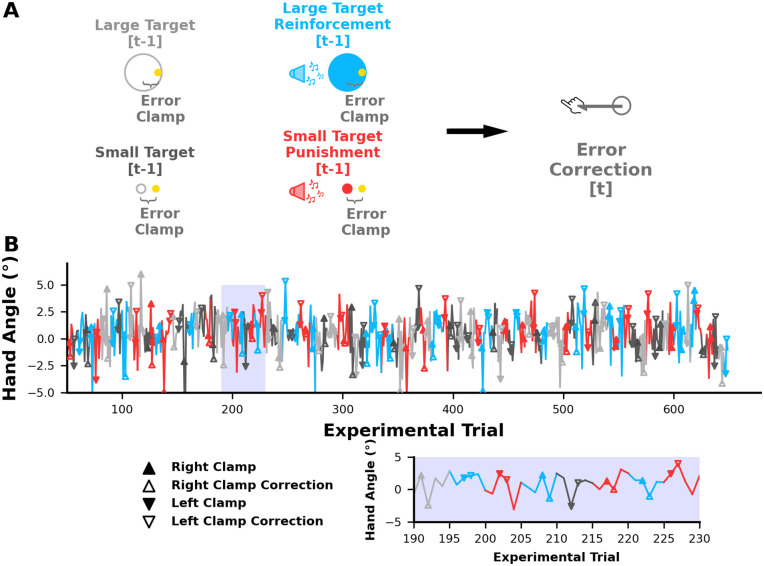
Experiment 3 Design and Individual Data. In Experiment 3, participants performed the reaching task with pseudorandom feedback conditions. To isolate implicit error corrections in Experiment 2, participants were instructed to maintain a constant explicit strategy, i.e., “always aim to the target center and ignore the cursor feedback”. **A)** Participants experienced interleaved mini-blocks with different feedback: a large target with endpoint cursor feedback (top left), a large target with reinforcement and endpoint cursor feedback (top right), a small target with endpoint cursor feedback (bottom left), or a small target with punishment and endpoint cursor feedback (bottom right). Critically, to study the immediate-history of reinforcement on error corrections (i.e., **[t]**), reinforcement or punishment feedback was provided on the same trial as pseudorandom error clamps (i.e., [t-1]). Further, to control for task errors, the error clamp was always within the target for both the large target and large target reinforcement conditions. Conversely, the error clamp was always outside the target for both the small target and small target punishment conditions. That is, for all large target conditions there was no task error and for all small target conditions there was always a task error, allowing us to observe the independent influence of reinforcement and punishment on error corrections. **B)** Hand angle (y-axis) over each trial (x-axis) of an individual participant. Light grey represents the large target trials, light blue the large target reinforcement trials, dark grey the small target trials, and red the small target punishment trials. To better illustrate the error clamps, corrections, and shuffling of the feedback conditions, the data shown within the purple shaded rectangle are expanded in the lower, purple shaded panel.

Participants experienced four different feedback conditions: large target, large target with reinforcement, small target, and small target with punishment (Fig 4). In all conditions, participants saw brief cursor feedback of their hand position when they passed by the target. In the large target with reinforcement condition, participants would receive reinforcement feedback if they hit the target. Similarly, in the small target with punishment condition, participants would receive punishment feedback if they missed the target. Participants experienced interleaved mini-blocks that probed the influence of reinforcement and punishment when given on the trial immediately before [i.e., t-1] an error correction [i.e., t]. Error clamps for all conditions were 2°, such that the cursor was embedded within the target for the large target condition and large target reinforcement condition. Conversely, the error clamp was always outside the target for the small target condition and small target punishment condition. Critically, keeping target sizes constant allowed us to test whether the immediate-history of reinforcement or punishment influenced implicit error corrections—independent of task errors [47,51,52].

#### Experiment 3 results.

In Experiment 3 we manipulated the immediate-history of reinforcement or punishment to test their influence on implicit error corrections. Fig 4B shows hand angle data from a representative participant illustrating how the feedback conditions were pseudorandomized and how participants responded to error clamps.

**Fig 5:**
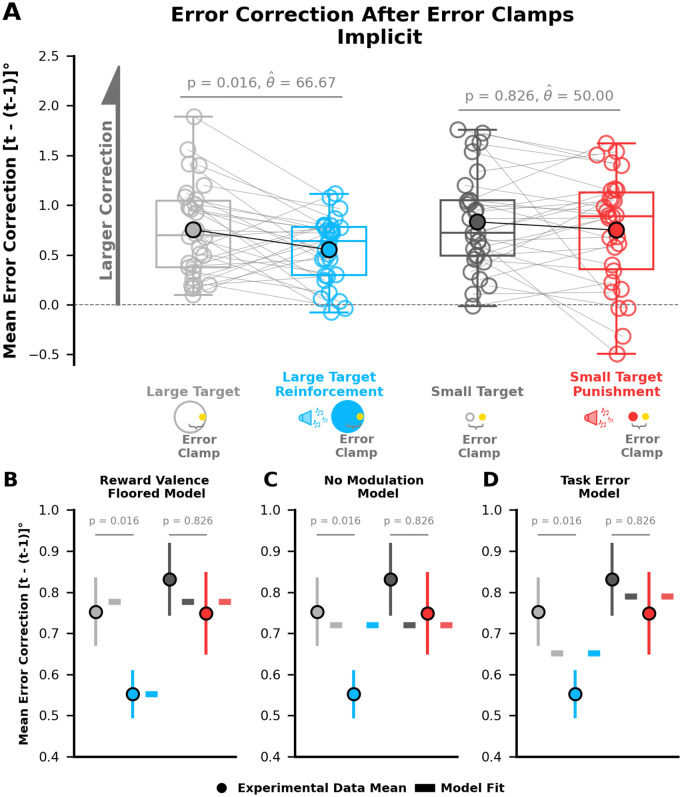
Experiment 3 Group Behaviour and Model Fitting. Mean error corrections (y-axis) for each feedback condition (x-axis). **A)** Error corrections to clamps were smaller in the large target reinforcement condition than the large target condition. There were no differences in error correction between the small target punishment and small target conditions. Solid circles are the group-level averages. Hollow circles are individual participants. Box and whisker plots are drawn for group data. **B)** The Reward Valence Floored model best captures the reinforcement modulation and lack of punishment modulation. Solid circles are the group-level averages and error bars show the standard error of the mean. Coloured small rectangles are model fits to experimental data, where the colour of the rectangle matches the experimental condition. Neither the **C)** No Modulation Model nor the **D)** Task Error Model can capture reinforcement modulation. Experiment 3 results support that the immediate-history of reinforcement modulates implicit error corrections.

We found that the presence of reinforcement led to a smaller error correction in the large target reinforcement condition compared to the large target condition (p = 0.016, $\hat{\theta }=66.67$). This finding aligns with the idea that an immediate-history of reinforcement modulates implicit error corrections (Fig 5A).

Conversely, we found no difference in error corrections between the small target condition and small target punishment condition (p = 0.826, $\hat{\theta }=50.00$). Thus, while we found that the presence of reinforcement led to smaller implicit error corrections, we did not see an influence of punishment on implicit error corrections.

#### Experiment 3 model - *a posteriori* fits.

In Experiment 3 we found that the immediate-history influenced error corrections. Thus, we considered the possibility that the immediately experienced reward valence may modulate implicit error corrections.

Next we modelled the computational effect of reward valence. That is, the received reward ($\alpha r_{t}$) directly modulates error corrections:


$$\begin{array}{c} X_{t+1}^{implicit}=X_{t}^{implicit}+\left(1-\alpha r_{t}\right)\beta^{implicit}(T-X_{t}^{implicit}) \end{array}$$ (10)

As a reminder, α is the reward valence, the signed reward magnitude, and $r_{t}$ is the presence of reward. The Reward Valence model is similar to Kim and colleagues’ (2019) Adaptation Modulation model [47]. The Reward Valence model would be able to capture the finding that the immediate-history of reinforcement would modulate implicit error corrections. However, the Reward Valence model would fail to capture our experimental finding that the immediate-history of punishment (i.e., negative valence) did not influence implicit error corrections.

A reasonable candidate model would need to capture both (1) a modulatory effect of reinforcement feedback and (2) no influence of negative reward valence, which is satisfied with the model below.

Reward Valence Floored Model:


$$\begin{array}{c} X_{t+1}^{implicit}=X_{t}^{implicit}+\left(1-\frac{|\alpha r_{t}|+\alpha r_{t}}{2}\right)\beta^{implicit}(T-X_{t}^{implicit}) \end{array}$$ (11)

Here the reward valence is floored because any time $\alpha r_{t}$ becomes negative, it is cancelled by its absolute value $\left|\alpha r_{t}\right|$. A floored reinforcement signal allows for positive reward to modulate error corrections, and floors aversive or punishing stimuli. Likewise, we also considered an Expected Value Floored Model and a Reward Prediction Error Floored Model (see Methods).

The Reward Valence Floored model (Fig 5B) outperforms the Expected Value Floored Model, the Reward Prediction Error Floored Model, and No Modulation Model (Table 1, **Experiment 3**) based on AIC and BIC. Furthermore, a supplementary analysis shows that the best fit Expected Value Floored and Reward Prediction Error Floored models converge to the Reward Valence Floored model (**Supplementary B** in S1 Appendix).

With our experimental design we were able to dissociate the influence of reinforcement and task errors. The Task Error Model [47] (see Methods) failed to capture our experimental finding where the immediate-history of reinforcement modulated implicit error correction (Fig 5D).

Taken together, our results suggest that a long-history of reinforcement modulates explicit error corrections and an immediate history of reinforcement modulates implicit error corrections.

## Discussion

Our results show that reinforcement history suppresses explicit and implicit error corrections. Our experiments and models suggest that a relatively higher expected value of a target decreases explicit error corrections. We also show that the reward valence from the previous trial reduces implicit error corrections.

Taken together, **Experiment 1** and **Experiment 2** results suggest that a more successful, long-history of reinforcement causes participants to make smaller explicit error corrections. Error corrections are composed of an explicit correction, an implicit correction, and any explicit-implicit interaction. In Experiment 2, we eliminated explicit corrections and therefore isolated implicit error corrections from either the explicit correction and any explicit-implicit interaction. We did not find any direct influence of the long-history or short-history of reinforcement on implicit corrections. Critically, this means the influence of the long-history of reinforcement in Experiment 1 must be a consequence of explicit error corrections. We initially considered that expected value or reward prediction error could modulate error corrections. Aligned with the expected value model, we found that a more successful long-history of reinforcement led to suppressed explicit error corrections. This suggests the utilization of expected value by the explicit system is a higher-level cognitive process and that same quantity is not influencing implicit error corrections. In **Experiment 3**, we manipulated the immediate-history of reinforcement or punishment to test their influence on implicit error corrections. We found that an immediate-history of reinforcement suppressed error corrections.

Our **Experiment 1, 2, & 3** results align with the findings of van der Kooij and colleagues (2018). During a virtual reality task, they provided participants both reinforcement and error feedback when attempting to hit a target. They found error corrections were smaller with a greater frequency of reinforcement feedback [42]. The authors suggested their result was from an implicit reinforcement process acting in opposition to error corrections. Our work suggests a complementary possibility, that their findings may also reflect reinforcement history suppressing error corrections.

Ours and van der Kooij (2018) results seemingly contradict Nikooyan and Ahmed (2015) [43], who found that reinforcement feedback increases error corrections against a constant visual rotation of feedback. This puzzling difference is resolved by examining experimental differences in reinforcement feedback. We and van der Kooij (2018) provided binary reinforcement feedback. On the other hand, Nikooyan and Ahmed (2015) gave graded reinforcement feedback, which increased in numeric value as participants ascended a gradient that aligned with the direction of error corrections. Unsurprisingly, humans ascend reinforcement gradients [27,43,53]. Graded reinforcement feedback can provide directionality to explicit strategies. There is an incentive to move further along the graded feedback to get to greater reward. Rather than measuring a modulatory effect of reinforcement on error corrections, Nikooyan and Ahmed (2015) likely measured how graded reinforcement can continuously shift explicit aiming towards a better strategy. Sugiyama and colleagues (2023) also used graded reinforcement feedback to shape error corrections. Unlike Nikooyan and Ahmed (2015) they found no effect of reinforcement feedback on adaptation learning rates, but they did find a difference in learning retention. That effects found in Nikooyan and Ahmed (2015) and Sugiyama (2023) is likely separate from the modulatory influence of reinforcement history we have identified in this work. How graded reinforcement feedback interacts with the modulatory effects of reinforcement history is an interesting and open question.

In Experiment 3, we showed that reward valence from the previous trial suppresses implicit error corrections. In contrast, Al-Fawakhiri and colleagues (2023) found no influence of reinforcement on implicit adaptation. Comparing the two studies, we had greater statistical power (N = 30 within subjects design vs. N = 24 between subjects design), and we gave both visual, monetary, and auditory reinforcement feedback instead of only auditory feedback. This inconsistency points to the potentially small effect of reinforcement on implicit error corrections.

Surprisingly, we found no influence of the immediate-history of punishment on implicit error corrections. Previous work by Galea and colleagues (2015) and Sugiyama and colleagues (2023) showed that participants learn more with greater punishment feedback [24,54]. In combination with our result, this would suggest that faster learning with punishment feedback is driven by explicit corrections, but aiming reports [8] are likely required to fully understand the influence of punishment feedback on explicit error corrections. Further work is needed to parse the explicit and implicit effects of punishment feedback.

An important aspect of our experimental design in Experiment 3 was controlling for the potential influence of task errors. Operationally, a task error is when a participant misses their motor goal [47,51,55]. Task errors are known to drive explicit action selection [7,8,15,26]. We controlled for explicit corrections in Experiment 3, and here were primarily interested in controlling the *implicit* effects of task error. In our task, we controlled for implicit task errors by embedding error clamps within a target in our large target and large target reinforcement conditions. Therefore, there was no implicit task error in either condition. This experimental control allowed us to probe the influence of reinforcement given immediately before an implicit error correction, without confounding it with an implicit task error signal.

Hitting a visual target (no task error) attenuates implicit error corrections [47,51,52]. Opinions in the literature are currently split on how implicit task error drives the attenuation of implicit sensorimotor adaptation. Some favour the idea that implicit task errors drive an independent learning process [47,52]. Conversely, others believe implicit task errors are just a moniker for reinforcement-based modulation [46]. Here we have shown that extrinsic reinforcement feedback suppresses implicit sensorimotor adaptation. Our effect is similar to others that show a target hit (no implicit task error) attenuates implicit error corrections. Therefore, our extrinsic reinforcement feedback may simply be a gain on a shared mechanism. Targets hits may generate an “intrinsic” reinforcement signal. In fact, evidence from zebra finches shows that dopaminergic neurons in the ventral tegmental area (VTA) respond to hitting or missing sensorimotor targets during song practice, supporting the existence of an “intrinsic” reinforcement signal [56,57]. Moreover, this activity in the VTA is causal. Lesioning the VTA impairs song practice in zebra finches even in the absence of extrinsic reinforcement [58]. Given the evidence for an intrinsic reinforcement signal, we question the parsimony of invoking an independent implicit task error process.

Previous models that consider the effects of reinforcement and error acting together have taken the same general approach. Specifically, both Izawa and Shadmehr (2011) and Roth and colleagues (2024) conceptualized error and reinforcement as two independent learning processes [59]. Cashaback (2017) showed that error-based processes dominated over reinforcement-based processes to update reach aim. Our followup work highlighted that these two independent processes can still have an interplay with one another, since they are both updating at the same time [30,31]. This work led us to the current study and modelling framework, where we tested the idea of whether the reinforcement-based process directly interacts with error-based processes. Therefore, we chose to model the connection between error and reinforcement as a modulatory relationship [24,47,54]. In **Experiment 1 & 2** we used the simplest modelling architecture possible that could capture error corrections and potential modifying quantities (e.g., expected value and reward prediction error). Similar to Galea et. al (2014) we used a single rate state space adaptation model, but also considered various modulatory terms on the error correction [24]. In Experiment 1 & 2, our modelling analysis supports that expected value modulates explicit error corrections. In **Experiment 3**, we followed a similar approach and considered modifying quantities on a single rate state space model. Our best fit model was the Reward Valence Floored model, which captured both (1) the immediate-history of reinforcement attenuating implicit error corrections and (2) punishment’s limited effect in Experiment 3. Together, these models suggest that reinforcement history influences error corrections through a higher-level explicit representation of expected value, and that a reinforcing stimuli during an error effects implicit error corrections through a lower-level mechanism acting through the reinforcer.

There are limitations to our simple modelling approach. Similar to Kim and colleagues’ (2019) Adaptation Modulation Model, reinforcement in all of our models is operationalized as binary [47,51]. However, reinforcement stimuli of different magnitude influence dopaminergic neurons in a continuous fashion [2]. How differences in reward magnitude (e.g., monetary value) influences error corrections is currently unknown. Furthermore, reinforcement of varying magnitude can be presented along gradients [27,43]. Future models will need to address the influence of reward magnitude and gradients on both explicit and implicit error corrections.

Neural evidence is consistent with midbrain dopaminergic neurons interacting with implicit and explicit error corrections. Converging evidence from neuroanatomy, rodent models, and clinical populations have associated motor learning with midbrain dopaminergic neurons, the neural substrate of the reward system [25,29,31,34,60–65]. The connections of dopaminergic neurons are sweeping, and one dopaminergic neuron may make up to one million synaptic connections [66]. These sprawling connections interface with multiple regions, including the dorsolateral prefrontal cortex [67,68] and cerebellum [69,70]. The dorsolateral prefrontal cortex is linked with explicit [71] error corrections and the cerebellum is associated with implicit error corrections [16,35,72–76]. These connections may be modulatory, as dopaminergic neurons potentiate memories at the cellular and behavioural level [67,68,77]. Modulatory connections between the motor and reward regions of the brain are consistent with our behavioural results.

Here we directly controlled reinforcement history, and parsed its effect on explicit and implicit error corrections. Future work could address the interactions between explicit and implicit corrections, potentially by directly manipulating explicit strategies. Taken together, our results show that a successful reinforcement history suppresses both explicit and implicit error corrections. Understanding the interactions between multiple motor learning processes has direct implications for all settings where motor skills are learned—from neurorehabilitation, athletics, prosthetics, and beyond.

## Methods

### Ethics statement

All participants provided written informed consent in accordance with the University of Delaware’s Institutional Review Board. The University of Delaware approved the proposed research and submitted documents via Full Committee Review in compliance with the pertinent federal regulations. The IRB approval number is 1602458.

### Participants

111 healthy adults were recruited across three experiments. Participants were free of all orthopaedic and neurological conditions that would influence reaching. Experiment 1 had 40 participants (28 Females and 12 Males, average age: 24±3.5 (standard deviation) years), Experiment 2 had 40 participants (17 Females and 23 Males, average age: 23±4.0 years), and Experiment 3 had 31 participants (16 Females and 15 Males, average age: 22±2.0 years). One participant’s data was excluded from Experiment 3 due to their inability to follow instructions (e.g., purposely closing eyes while reaching to targets). Participants were informed they would receive a base compensation of $5.00 during the experiment with up to an additional $5.00 based on their performance. All participants received the full $10.00 independently of their performance.

### Apparatus

All experiments were performed on a KINARM endpoint robotic manipulandum (Fig 1A; BKIN Technologies, Kingston, ON). Each participant grasped the end of the manipulandum with their right hand and made reaching movements in the horizontal plane. A semi-silvered mirror blocked the vision of their arm and hand, while also reflecting virtual images (e.g., targets, cursors) from an LCD into the horizontal plane of the motion. All tasks were custom coded in MATLAB Simulink, and then compiled in C to be run on KINARM’s Dexterit-E software. Kinematic data were recorded at 1000 Hz and stored offline for analysis. All data were de-identified and imported onto an external personal computer for analysis.

### Experimental design

#### General task protocol.

In all experiments, participants saw a circular home location (0.75 cm radius). The home location was 15 cm from each participant’s sternum. In all experiments, participants also saw a singular, circular target. For Experiment 1 and 2 the target had a radius 1.25 cm (Fig 1A). For Experiment 3, the target was small or large depending on the trial. The targets had a radius of 0.5 cm and 1.25 cm, respectively (Fig 4A). The forward distance from the home location to the center of the target was 20 cm. In all experiments, participants also saw a thin boundary arc. The thin boundary arc had a 0.5 cm width and 90° angular size (Fig 1A) The forward distance from the home location to the boundary arc was 23.25 cm.

At the start of each trial, the robotic manipulandum would move their hand to the home location along a minimum jerk trajectory. When within 1.6 cm of the home location, the participants could see a yellow circular cursor (0.3 cm radius) aligned with their hand. Once their hand stopped within the home location, participants waited between 250–1000 ms drawn from a uniform distribution. The home location then turned yellow to indicate that the participant was free to initiate a reach towards the target. Participants were verbally encouraged to initiate a reach at the presentation of the go cue. If a participant initiated a reach too early they were verbally instructed to move back to the home location and wait until it turned yellow. Participants were instructed to cross over the boundary arc and stop. The boundary arc disappeared once crossed. The trial ended once their hand was stationary for 125 ms. The purpose of this procedure was to prevent anticipatory, erroneous reaches. In **Supplementary D** in the S1 Appendix, we analyze average response times of participants and show there was no influence of response time on mean error corrections for Experiment 1 and 2. That analysis supports response time did not effect the relative engagement of the explicit versus implicit process.

### Types of task feedback.

#### Reinforcement Feedback.

When participants were given extrinsic reinforcement feedback the target briefly turned blue and expanded, they heard a pleasant noise, and participants were told they earned monetary reward towards a performance bonus. Past research shows visual, auditory, and monetary stimuli to be reinforcing [78–80].

#### Punishment Feedback.

In just Experiment 3, participants could receive punishment feedback. Participants saw the target turn red, they heard a negative buzzer sound, and were told they lost money from a performance bonus.

#### Error Clamp.

When given an error clamp [48], participants were briefly shown endpoint cursor feedback when they passed through the horizontal axis of the target. Endpoint feedback has been used effectively to induce implicit and explicit error corrections in trials following an error [7,8,15,81]. Endpoint feedback also prevents online feedback error corrections, which ensures our metrics were measuring feedforward motor commands.

Unknown to the participants, however, the cursor feedback was experimentally controlled to be a set distance from the center of the target. We experimentally controlled the error size to assess participants’ error corrections on the next trial.

#### Veridical Endpoint Feedback.

When given veridical endpoint feedback, participants had their cursor position briefly flashed at their hand location when they passed through the horizontal axis of the target.

**No Feedback.** Finally, participants could also receive no visual feedback of their performance.

#### Experiment 1 and 2 design.

The goal of Experiment 1 was to manipulate the reinforcement history to test whether expected value or reward prediction errors modulates explicit and/or implicit error corrections. In this experiment, participants were instructed to “hit the target” and were free to explicitly direct their aim.

The goal of Experiment 2 was to manipulate the reinforcement history to test whether expected value or reward prediction errors modulate just *implicit* error corrections. In contrast to Experiment 1, participants were instructed to “always aim to the target center and ignore the cursor feedback” for all trials. Participants were also educated on explicit re-aiming strategies using a dartboard example on a whiteboard. The experimenter communicated that performance at darts can improve by changing your aiming location, or by “subconsciously” improving. The experimenter told participants we were interested in this “subconscious” component of learning and therefore to always aim for the target center. Participants repeated the instructions back to the experimenter to confirm they understood the task. This instruction allowed us to isolate implicit error corrections from any contaminating effect of explicit corrections. Apart from any instruction differences, all aspects of Experiment 1 and 2 used the same experimental design.

#### Long-history of reinforcement.

The long-history of reinforcement was manipulated by providing probabilistic reinforcement feedback across the majority of the trials. Participants were randomized into a 20% or an 80% probability of reinforcement group. A consequence of this probabilistic methodology is that any individual would not necessarily receive an actual reward rate of 20% or 80%. Critically, these different reinforcement probabilities acting over many trials allowed us to dissociate whether the sensorimotor system uses expected value or reward prediction errors to modulate error corrections.

For both Experiments 1 and 2, participants performed 50 baseline trials. Participants received endpoint feedback in the first 30 trials of baseline. Participants received no visual feedback in the last 20 trials of baseline.

After baseline, participants performed 48 blocks that each contained 13 trials (Fig 1D). For the first 10 trials of each 13 trial block [i.e., t-13,...,t-4], participants had either a 20% or an 80% probability of reinforcement feedback when they reached through an unseen reward region. Otherwise, participants received no feedback.

Each participant’s baseline variability was used to calculate the size of the unseen reward region. Baseline variability was calculated over the last 40 baseline trials. The last 40 trials contained a mixture of visual (first 20 trials) and no visual feedback trials (last 20 trials). This mixture is representative our task, given it was a mix of visual and no visual feedback.

The region was centered on the target and its width was ±3 standard deviations of participant baseline variability [27]. The large but finite size of the reward region prevented any obvious misses from being rewarded. This also helped keep participants naive to the reinforcement feedback manipulation.

#### Short-history of reinforcement.

The short-history of reinforcement was manipulated by using reinforcement clamps (Fig 1D). These reinforcement clamps were provided to the participant after the first 10 trials of each block [i.e., t-3, t-2]. Specifically, we manipulated whether they experienced a successful short-history [t-3 = reinforcement, t-2 = reinforcement] or a less successful one [t-3 = reinforcement, t-2 = no reinforcement]. Participants received only binary reinforcement feedback and no endpoint feedback on short-history reinforcement clamps. Half of the short-history clamps were a successful short-history and half were a less successful short-history. The order of these clamps were pseudorandomized within “super-blocks” of 8 clamps. Our pseudorandomization prevented the successive presentation of too many of the same short-history clamp type. This manipulation allowed us to probe any short term effects of reinforcement on upcoming error corrections.

#### Error Clamps.

The last trial [t-1] of every block was an error clamp (Fig 1C). Error clamp locations could be in the center or outside of the target. The error clamp location was pseudo-randomized between the blocks.

Half of the error clamps (24 out of 48) imposed a visual error on the cursor of 4.4°. Error clamps were relatively small for a few reasons. One was to protect subjects’ sense of contingency. Larger errors are more noticeable [82]. Secondly, we wanted to limit any potentially dominating effects of explicit corrections. The explicit process can compensate with greater magnitude and more quickly to large errors [83,84]. Also, implicit corrections peak at error sensitivity at smaller error sizes [85]. Therefore, smaller clamp sizes ensured a more equal contribution of both processes. The direction of these imposed errors was counterbalanced so that half of these error clamps were on the left and the other half on the right side of the target. No reinforcement feedback was given on missed trials.

Half of the error clamps (24 out of 48) imposed a visual error on the cursor of 4.4°. Error clamps were relatively small for a few reasons. One was to protect subjects’ sense of contingency. Larger errors are more noticeable [82]. Secondly, we wanted to limit any potentially dominating effects of explicit corrections. The explicit process can compensate with greater magnitude and more quickly to large errors [83,84]. Also, implicit corrections have the greatest error sensitivity at small error sizes [85]. Therefore, smaller clamp sizes ensured a more equal contribution of both processes by preventing large explicit corrections and ensuring the implicit system was still sensitive to the error. The direction of these imposed errors was counterbalanced so that half of these error clamps were on the left and the other half on the right side of the target. No reinforcement feedback was given on missed trials.

The position for the other half of error clamps was randomly drawn from a uniform distribution. The distribution spanned half the width of the target aligned on the target center. These errors clamps within the target helped keep participants naive to the feedback manipulation by simulating a range of successful trials, while still controlling their actual feedback. Participants also received reinforcement feedback on the center clamp trials as they hit the target.

Our pseudorandomized procedure for error clamp types also contributed to participant naivety. We organized every 8 error clamps into a “super-block.” In each super block we had 2 left clamps, 2 right clamps, and 4 clamps within the target center. Our pseudorandomization prevented the successive presentation of more than two of the same clamp type.

After an error clamp trial, participants corrected for the error clamp on the next trial [t]. Trial [t] was the first trial of the next block.Error corrections to right error clamps were multiplied by -1 so that corrections to both right and left clamps were expressed as positive values. The mean error correction was taken across all error corrections as the main dependent measure for each participant.

#### Experiment 3 design.

The goal of Experiment 3 was to test whether the immediate-history of reinforcement or punishment influences implicit error corrections. Participants had identical instructions to Experiment 2 (“always aim to the target center and ignore the cursor feedback”) and were also instructed on the nature of explicit re-aiming.

Immediate-history of reinforcement and punishment. The immediate-history of reinforcement was manipulated by providing reinforcement on the same trial as error clamps [i.e., t-1]. Likewise, the immediate-history of punishment was manipulated by providing punishment on the same trial as error clamps [i.e., t-1].

We used a within subjects design where participants experienced one of four possible conditions: large target, large target with reinforcement, small target, and small target with punishment (Fig 4A). In the large target condition, participants received no reinforcement. In the large reinforcement condition, participants received reinforcement when they hit the target. Notably, they also received reinforcement during error clamp trials. In the small target condition, participants did not receive punishment when they missed the target. In the small target punishment condition, participants received punishment when they missed the target. Participants completed 7-point Likert scales to verify that reinforcement and punishment feedback were interpreted correctly. They responded to the statements, “It felt good when I hit the target and it turned blue” and “It felt bad when I missed the target and it turned red”. On average participants agreed with both statements (average score for reward: 6.58, average score for punishment: 6.03). They also received punishment during error clamp trials. All conditions showed participants endpoint feedback, although it was not veridical during error clamps.

Baseline trials for each individual were identical to Experiment 1 and 2. Participants experienced 15 blocks of 40 trials. Each block was subdivided into 8 mini-blocks of 5 trials, presented in pseudorandom order (Fig 4B). Each mini-block was only one of four conditions. During the middle 3 trials of each mini-block, one of the trials was randomly chosen to be an error clamp. This mini-block structure ensured that the error clamps and error corrections were within the same condition. Each condition appeared twice within a block to counterbalance the error clamps on both sides of the target.

Error clamps [i.e., t-1] for all conditions were 2°. Thus, the cursor feedback was embedded within the target for the large target and large target reinforcement conditions. Keeping a constant target size between the large target and large target reinforcement conditions allowed us to test whether reinforcement influenced implicit error corrections, independent of task errors [47,52]. Conversely, the error clamp was always outside the target for the small target and small target punishment conditions. Keeping a constant target size between the small target and small target reinforcement conditions allowed us to test whether punishment influenced implicit error corrections, independent of task errors. That is, for all large target conditions there was no task error and for all small target conditions there was always a task error, allowing us to observe the independent influence of reinforcement and punishment on error corrections. After an error clamp trial, participants corrected for the error clamp on the next trial [i.e., t].

### Data analysis

Data analysis was performed using Python. Endpoint was measured when the hand position crossed the y-position of the target. Reach angle was calculated as the angle between 1) the line that intersected the home location and target, and 2) the line that intersected the home location and endpoint. Reach angles were calculated as positive when clockwise relative to the target, with the home location as the center of rotation. Error corrections were calculated as the hand angle difference between trial after and on an error clamp. Error corrections to right error clamps were multiplied by -1 so that corrections to both right and left clamps were expressed as positive values. The mean error correction was taken across all error corrections as the primary outcome measure for each participant.

### Statistical analysis

All statistical tests were performed using the Python Pingouin package unless otherwise stated. A 2 (80% and 20% Reinforcement) x 2 (Reinforcement Clamp 1 and 2) mixed ANOVA was performed for both experiment 1 & 2 as an omnibus test. Follow-up mean comparisons were performed with bootstrapped permutation tests (1,000,000 bootstraps) [86–89] using custom functions in the Cashaback Lab’s analysis utilities package (https://github.com/CashabackLab/AnalysisUtilities). All permutation tests were Holm-Bonferroni corrected to control the type 1 error rate. The significance level was set to α=0.05. Two-tailed tests were used in Experiment 1 and 2 because of no clear directionality in the theoretical predictions. In Experiment 3, one-tailed tests were used because of expected theoretical effects. The results and interpretation remained the same for one and two-tailed tests. We computed the Common Language Effect Size ($\hat{\theta }$) for all mean comparisons.

## Computational modelling

### Experiment 1 models.

The sensorimotor system corrects movements after errors. A first order approximation of this process is the single rate state space model [13,20,49,50]. Here we consider the simple case without motor noise:


$$\begin{array}{ccc} e_{t} & = & \left(T-X_{t}\right) \end{array}$$ (12)

$$\begin{array}{ccc} X_{t+1} & = & X_{t}+\beta e_{t} \end{array}$$ (13)

where $e_{t}$ is the error signal between the motor target (*T*) and the sensory feedback of the executed movement ($X_{t}$). β is the learning rate on the error signal. $X_{t+1}$ represents the adjustment of the motor system (i.e., change in an internal model or action selection).

We consider that the reinforcement system may modulate error corrections. This modulation may arise from one of two key quantities encoded in the midbrain dopaminergic neurons: expected value or reward prediction error [2,36–38].

### Expected value and reward prediction error calculations

Here, we model the estimation of expected value and the calculation of reward prediction error from the environment with a Monte Carlo reinforcement model (Sutton & Barto, eq. 6.1) [1,90–93]. In a Monte Carlo reinforcement model, the agent samples states and reward is received at the end of each trial. The total reward ($\alpha r_{t}$) is then used to update a value function (*EV*, expected value). Here, the value function for each state *T* (i.e., a motor target) is updated by the reward prediction error ($RPE_{t}$). RPE is the difference between actual ($\alpha r_{t}$) and expected reward ($EV_{t}(T)$), multiplied by a learning rate (γ) at the end of each trial:


$$\begin{array}{ccc} RPE_{t} & = & \alpha r_{t}-EV_{t}(T) \end{array}$$ (14)

$$\begin{array}{ccc} EV_{t+1}(T) & = & EV_{t}(T)+\gamma RPE_{t} \end{array}$$ (15)

where $r_{t}$ is the signed presence of reward ($r_{t}=1$), absence ($r_{t}=0$), or punishment ($r_{t}=-1)$. In our simple experiments, the agent only has one possible *T*. Thus, we will drop the state argument from future notation for simplicity. Using this framework we develop hypotheses for single trial error corrections.

Expected Value Model: One hypothesis is that expected value directly modulates the error correction in the single rate state space model:


$$\begin{array}{c} X_{t+1}=X_{t}+\left(1-EV_{t+1}\right)\beta e_{t} \end{array}$$ (16)

Reward Prediction Error Model: Alternatively, another hypothesis is that Reward Prediction Error performs the modulation instead:


$$\begin{array}{c} X_{t+1}=X_{t}+\left(1-RPE_{t}\right)\beta e_{t} \end{array}$$ (17)

No Modulation Model: Finally, there may be no modulation of error corrections by either reinforcement learning quantity:


$$\begin{array}{c} X_{t+1}=X_{t}+\beta e_{t} \end{array}$$ (18)

Experiment 1 is agnostic to the explicit and implicit distinction based on the instructions to, “hit the target and ignore cursor feedback.” Likewise the models above aggregate implicit and explicit processes. The goal of Experiment 2 was to isolate the implicit contribution of error corrections.

#### Experiment 2 models.

The single rate state space model is a simplification of human sensorimotor adaptation. Previous work supports that error-based learning is composed of an explicit and implicit process [7,8,15]. Explicit strategies may be implemented nonlinearly, but we assume here that explicit corrections also follow a linear state space equation as in Taylor and colleagues (2011). For simplicity, we assume no complex interactions between the processes [94]. Implicit error corrections are calculated between the explicit motor target and the last executed movement, as in previous aim point correction models [27,31,49,95]. Overall motor execution ($X_{t}$) is a function of both the explicit and the implicit process: [83]


$$\begin{array}{ccc} X_{t+1}^{explicit} & = & X_{t}^{explicit}+\beta^{explicit}(T-X_{t}) \end{array}$$ (19)

$$\begin{array}{ccc} X_{t+1}^{implicit} & = & X_{t}^{implicit}+\beta^{implicit}(X_{t}^{explicit}-X_{t}) \end{array}$$ (20)

$$\begin{array}{ccc} X_{t+1} & = & X_{t+1}^{explicit}+X_{t+1}^{implicit} \end{array}$$ (21)

If we fix the explicit strategy to the motor target location (as in Experiment 2) for all *t* to zero [i.e., $X_{t}^{explicit}=T=0$], then we recover a single rate state space equation describing only the implicit process:


$$\begin{array}{c} X_{t+1}^{implicit}=X_{t+1}^{implicit}+\beta^{implicit}(T-X_{t}^{implicit}) \end{array}$$ (22)

Similarly, we consider whether expected value or the reward prediction error could modulate implicit error corrections in the following models.

Expected Value Model:


$$\begin{array}{c} X_{t+1}^{implicit}=X_{t}^{implicit}+\left(1-EV_{t+1}\right)\beta^{implicit}(T-X_{t}^{implicit}) \end{array}$$ (23)

Reward Prediction Error Model:


$$\begin{array}{c} X_{t+1}^{implicit}=X_{t}^{implicit}+\left(1-RPE_{t}\right)\beta^{implicit}(T-X_{t}^{implicit}) \end{array}$$ (24)

No Modulation Model:


$$\begin{array}{c} X_{t+1}^{implicit}=X_{t}^{implicit}+\beta^{implicit}(T-X_{t}^{implicit}) \end{array}$$ (25)

#### Experiment 3 models.

Reward Valence Model: The reward valence, $\alpha r_{t}$, directly modulates implicit error corrections [47].


$$\begin{array}{c} X_{t+1}^{implicit}=X_{t}^{implicit}+\left(1-\alpha r_{t}\right)\beta^{implicit}(T-X_{t}^{implicit}) \end{array}$$ (26)

The Expected Value and Reward Prediction Error models can become mathematically equivalent to the Reward Valence Model when only considering the *immediate* history of reinforcement (**Supplementary B** in S1 Appendix). For the Expected Value model this occurs when the learning rate of *EV*, γ, is ≈1. For the Reward Prediction Model this occurs when γ≈0. Indeed, in our modelling analysis both floored versions of the models converged to the reward valence model.

Reward Valence Floored Model: It is possible that actions with negative valence (punishment) do not or have little influence on error corrections. We implement this idea by flooring the reward valence in the reward valence model:


$$\begin{array}{c} X_{t+1}^{implicit}=X_{t}^{implicit}+(1-\frac{|\alpha r_{t}|+\alpha r_{t}}{2})\beta^{implicit}(T-X_{t}^{implicit}) \end{array}$$ (27)

Here the reward valence is floored because any time $\alpha r_{t}$ becomes negative, it is cancelled by its absolute value $\left|\alpha r_{t}\right|$.

Expected Value Floored Model:


$$\begin{array}{c} X_{t+1}^{implicit}=X_{t}^{implicit}+\left(1-\frac{|EV_{t+1}|+EV_{t+1}}{2}\right)\beta^{implicit}(T-X_{t}^{implicit}) \end{array}$$ (28)

Reward Prediction Error Floored Model:


$$\begin{array}{c} X_{t+1}^{implicit}=X_{t}^{implicit}+\left(1-\frac{|RPE_{t}|+RPE_{t}}{2}\right)\beta^{implicit}(T-X_{t}^{implicit}) \end{array}$$ (29)

No Modulation Model:


$$\begin{array}{c} X_{t+1}^{implicit}=X_{t}^{implicit}+\beta^{implicit}(T-X_{t}^{implicit}) \end{array}$$ (30)

Task Error Model: [47] Another possibility is that implicit error corrections driven by sensory prediction errors (SPE) occur alongside another implicit task error (TE) signal in a dual error model. Task error is the difference in desired motor performance (hitting the target) and actual motor performance.


$$\begin{array}{ccc} X_{t+1}^{SPE} & = & X_{t}^{SPE}+\beta^{SPE}(X_{t}^{explicit}-X_{t}) \end{array}$$ (31)

$$\begin{array}{ccc} X_{t+1}^{TE} & = & X_{t}^{TE}+\beta^{TE}(G-T_{t}) \end{array}$$ (32)

$$\begin{array}{ccc} X_{t+1}^{implicit} & = & X_{t+1}^{SPE}+X_{t+1}^{TE} \end{array}$$ (33)

Identical to Kim (2019), we operationalize the task error into a binary signal that is present with a target hit and absent with a target miss. As before, we fix the explicit process on the motor target, *T*.


$$\begin{array}{ccc} X_{t+1}^{SPE} & = & X_{t}^{SPE}+\beta^{SPE}(T-X_{t}^{implicit}) \end{array}$$ (34)

$$\begin{array}{ccc} TE & = & \left\{ \begin{array}{cc} 0, & \text{hit}\\ 1, & \text{miss} \end{array}\right. \end{array}$$ (35)

$$\begin{array}{ccc} X_{t+1}^{TE} & = & X_{t}^{TE}+\beta^{TE}TE \end{array}$$ (36)

$$\begin{array}{ccc} X_{t+1}^{implicit} & = & X_{t+1}^{SPE}+X_{t+1}^{TE} \end{array}$$ (37)

#### Model fitting.

Models were fit separately to each experiment. Procedures are similar to Roth (2024) [31,96]. Models were fit using the Powell algorithm in the optimize.minimize function from the Scipy Python package (v1.11.1).

Each model was fit by first simulating 500 participants. Given the probabilistic nature of our experimental tasks these multiple simulations ensured stable estimates of model outcomes. Different simulated participants had differing trial ordering and reinforcement. The Scipy optimizer was configured to minimize the mean squared error (MSE), using Powell’s method, between the subjects in each experimental group and the model, separately for each experiment.

Model fits began with a “warm-start” procedure where models were initialized with random parameters 5,000 times and then fit. The lowest loss parameters of the warm-starts were chosen as the initial parameters for our bootstrapping procedure. In that procedure our experimental data was resampled with replacement 10,000 times. Models were fit to the resampled data. Our bootstrapping procedure generated posterior distributions of the best fit parameters. These posterior distributions enabled us to generate 95% confidence intervals for each parameter (**Supplementary A** in S1 Appendix). The median of each parameter posterior distribution was used as the best fit parameter for each parameter and for each model. Reinforcement valence parameters (α) were constrained to be greater than 0 as the sign of the valence was hardcoded into $r_{t}$ in the simulations. β and γ were constrained to be between [0,1] as negative learning rates and learning rates greater than the error size were assumed to be erroneous.

#### Best-fit model selection.

Our model loss was defined as the mean-squared error (MSE) between the model prediction ($\hat{z}$) and each individuals’ average error correction (${\bar{x}}_{i,j}$):


$$\begin{array}{c} MSE=\sum_{i=1}^{M}\sum_{j=1}^{N}\frac{({\bar{x}}_{i,j}-{\hat{z}}_{i}{)}^{2}}{N} \end{array}$$ (38)

where M is the number of groups/conditions in a given experiment, and N is the number of participants in the given group/condition. The best fit models were first selected based on their ability to capture statistically significant experimental trends. A model was falsified if it could not explain the experimental result. If the model captured the experimental data, we then considered its Akaike Information Criteria (AIC) and Bayesian Information Criteria (BIC) score. AIC and BIC both score models based on Mean Squared Error (MSE), while punishing for the number of parameters. Smaller scores indicate relatively better models. The AIC and BIC can be calculated using MSE: [97]


$$\begin{array}{ccc} AIC & = & 2k+n\ln(MSE) \end{array}$$ (39)

$$\begin{array}{ccc} BIC & = & k\ln(n)+n\ln(MSE) \end{array}$$ (40)

## Supporting information

S1 Appendix Supplementary A: All Model Fits, Supplementary B: Convergence of Expected Value and Reward Prediction Error in Experiment 3, Supplementary C: Error Corrections over Time in Experiment 1 and 2, Supplementary D: Influence of Response Time on Error Corrections. (PDF)
