# Supplementary material for: Successful reinforcement history suppresses explicit and implicit error corrections
Source: PLoS Comput Biol. 2026 Aug 3;22(8):e1014574. doi: 10.1371/journal.pcbi.1014574 (PMC13432137; doi:10.1371/journal.pcbi.1014574)
Supplement: S1 Appendix — (PDF) [file pcbi.1014574.s001.pdf]

Supplementary A: All Model Fits

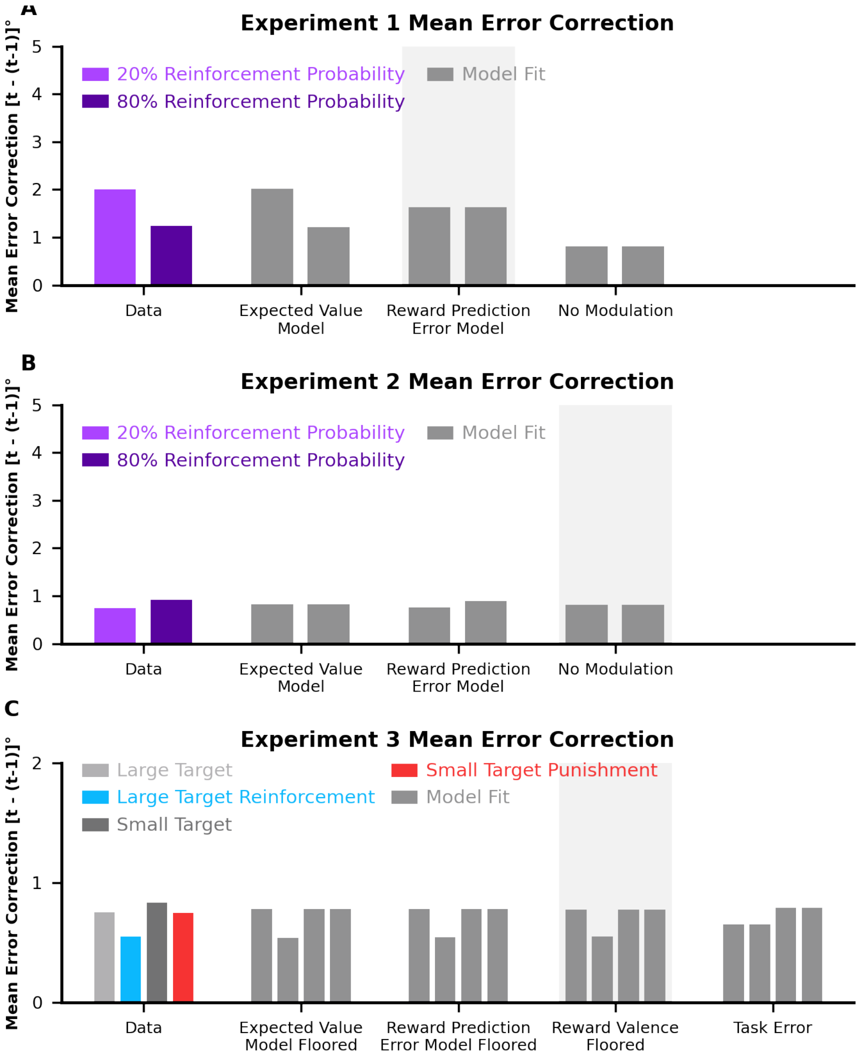

Supplementary Figure 1: Best Fit Model Results for Mean Error. 500 participants were simulated using the best fit parameters from our model fitting procedure. Mean error correction (y-axis) is plotted against each experimental group for each model (x-axis). Highlighted models are the the best fit models. These models best capture experimental trends, and minimize the relative Akaike and Bayesian Information Criterion between models. **A)** Simulations of Experiment 1. Colored bars are the experimental data and grey bars are the model simulations. **B)** Simulations of Experiment 2. Colored bars are the experimental data and grey bars are the model simulations. **C)** Simulations of Experiment 3. Colored bars are the experimental data and grey bars are the model simulations.

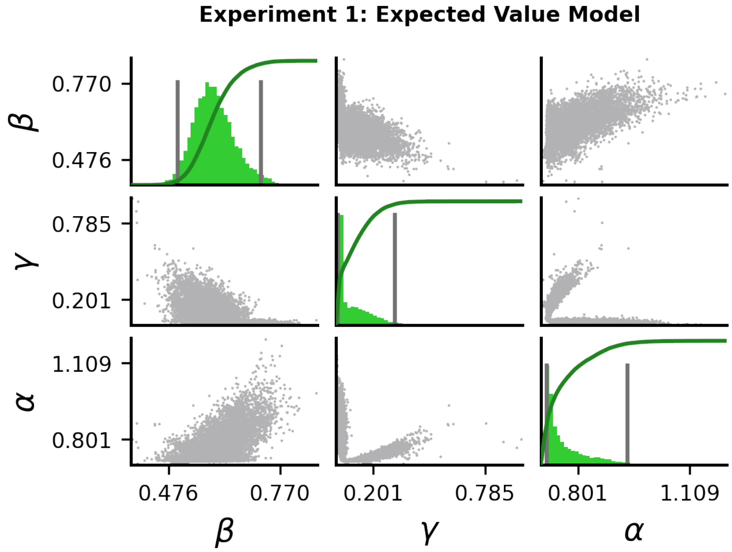

Supplementary Figure 2: Posterior Parameter Estimates of Expected Value Model in Experiment 1. Shown is a pair plot of the bootstrapped (10,000 samples) posterior parameter estimates. The diagonal plots contain the histograms of the posterior parameter distribution of each parameter in the model. The median parameters of the posteriors are used as the best-fit parameter estimates ($\beta = .592, \gamma = 0.050, \alpha = 0.742$). The off diagonal plots show joint distributions for each pair of parameters (y-axis and x-axis). 95% confidence intervals are indicated with dark grey lines. Thick dark green lines correspond to the cumulative distribution.

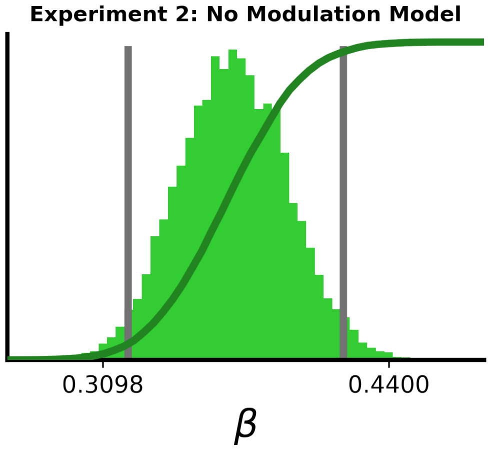

Supplementary Figure 3: Posterior Parameter Estimate of No Modulation Model in Experiment 1. Shown is a plot of the bootstrapped (10,000 samples) posterior parameter estimate for β. The median β of the posterior is used as the best-fit parameter estimate ($\beta = .184$). The 95% confidence interval is indicated with dark grey lines. The thick dark green line corresponds to the cumulative distribution.

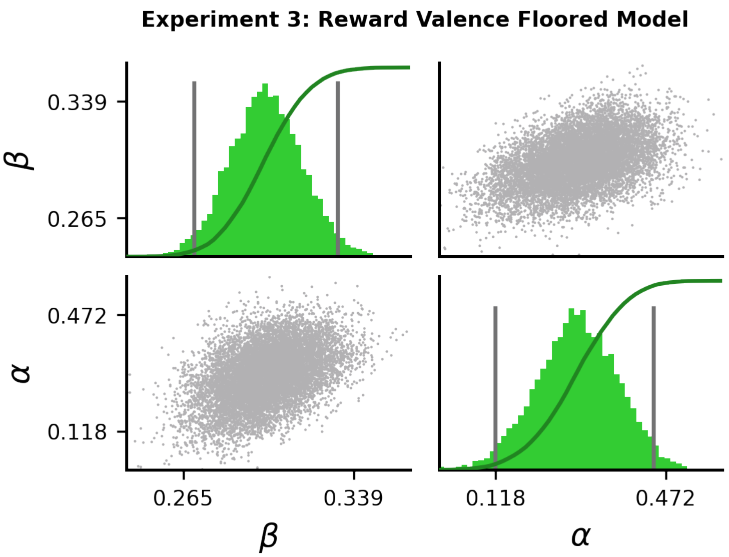

Supplementary Figure 4: Posterior Parameter Estimates of Reward Valence Floored Model in Experiment 1. Shown is a pair plot of the bootstrapped (10,000 samples) posterior parameter estimates. The diagonal plots contains the histograms of the posterior parameter distribution of each parameter in the model. The median parameters of the posteriors are used as the best-fit parameter estimates ($\beta = .301, \alpha = 0.289$). The off diagonal plots show joint distributions for each pair of parameters (y-axis and x-axis). 95% confidence intervals are indicated with dark grey lines. Thick dark green lines correspond to the cumulative distribution.

Supplementary B: Convergence of Expected Value and Reward Prediction Error in Experiment 3

Here we show that the Expected Value Floored and Reward Prediction Error Floored models can become mathematically equivalent to the Reward Valence Floored Model when only considering the immediate history. Our model fitting procedure shows the best fit parameters of these models converge in the following way, as shown in **Supplementary Figure 5 and 6**. Writing out the Expected Value Floored and Reward Prediction Error Floored Model:**thoroughman_learning_2000**

$$X_{t+1}^{implicit} = X_{t+1}^{implicit} + \beta^{implicit}(T - X_t^{implicit}) \tag{1}$$

$$e_t = (T - X_t) \tag{2}$$

$$RPE_t = \alpha r_t - EV_t \tag{3}$$

$$EV_{t+1} = EV_t + \gamma RPE_t \tag{4}$$

Expected Value Floored Model:

$$X_{t+1}^{implicit} = X_t^{implicit} + \left(\mathbf{1} - \frac{|\boldsymbol{EV_{t+1}}| + \boldsymbol{EV_{t+1}}}{\mathbf{2}}\right)\beta^{implicit}(T - X_t^{implicit}) \tag{5}$$

Reward Prediction Error Floored Model:

$$X_{t+1}^{implicit} = X_t^{implicit} + \left(\mathbf{1} - \frac{|\boldsymbol{RPE_t}| + \boldsymbol{RPE_t}}{\mathbf{2}}\right)\beta^{implicit}(T - X_t^{implicit}) \tag{6}$$

If the Expected Value model was maximally sensitive to the immediate history of reinforcement, the learning rate on expected value will approach $\gamma = 1$. Expected Value, in this case, is updated incredibly quickly. Making this substitution yields:

$$EV_{t+1} = EV_t + 1 * RPE_t \tag{7}$$

substituting in RPE

$$EV_{t+1} = EV_t + \alpha r_t - EV_t \tag{8}$$

and then substituting our new expression for EV_{t+1} into Eq. (45) yields:

$$X_{t+1}^{implicit} = X_t^{implicit} + \left(\mathbf{1 - \frac{|\boldsymbol{\alpha r_t}| + \boldsymbol{\alpha r_t}}{2}}\right)\beta^{implicit}(T - X_t^{implicit}) \tag{9}$$

If the Reward Prediction Error model was maximally sensitive to the immediate history of reinforcement, the learning rate on expected value will approach $\gamma = 0$, i.e. the reward prediction error does not consider historical information contained in the expected value. In that case, no past information would affect the reward prediction error. If EV is initialized at 0 ($EV_{t_0} = 0$), making the substitution yields:

$$EV_t = EV_{t_0} + 0 \tag{10}$$

$$EV_t = 0 \tag{11}$$

which is true for all t. This means RPE_t is

$$RPE_t = \alpha r_t \tag{12}$$

and substituting into Eq. (46) yields

$$X_{t+1}^{implicit} = X_t^{implicit} + \left(\mathbf{1 - \frac{|\boldsymbol{\alpha r_t}| + \boldsymbol{\alpha r_t}}{2}}\right)\beta^{implicit}(T - X_t^{implicit}) \tag{13}$$

which is identical to the Reward Valence Floored Model, as required.

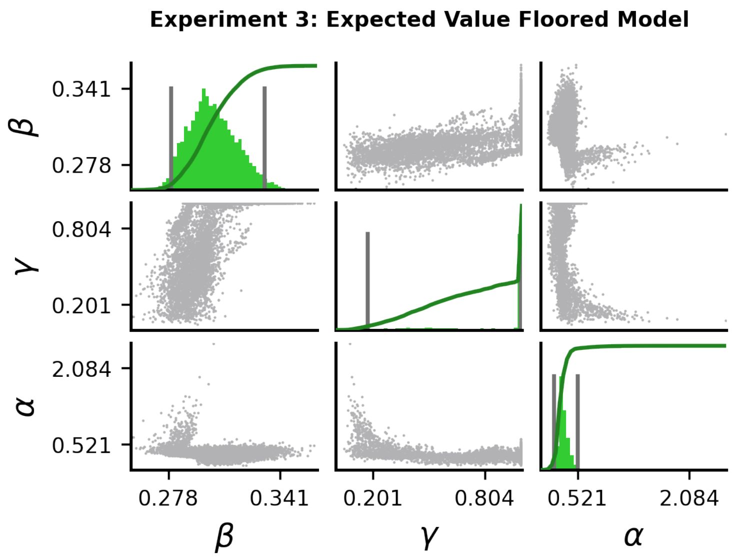

Supplementary Figure 5: Posterior Parameter Estimates of Expected Value Floored Model. Shown is a pair plot of the bootstrapped (10,000 samples) posterior parameter estimates. The diagonal plots contains the histograms of the posterior parameter distribution of each parameter in the model. Importantly, the best (median value of the posterior) estimate of γ is ≈ 1. This means the Expected Value Floored Model converges to the Reward Valence Floored Model. The off diagonal plots show joint distributions for each pair of parameters (y-axis and x-axis). 95% confidence intervals are indicated with dark grey lines. Thick dark green lines correspond to the cumulative distribution.

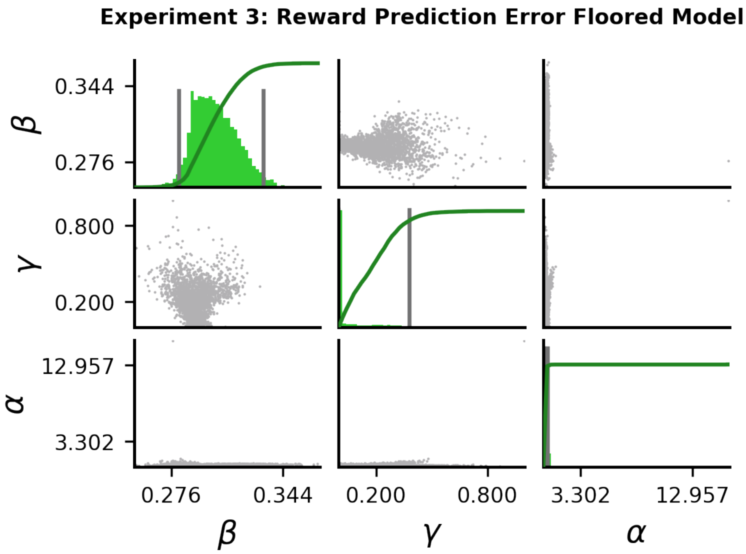

Supplementary Figure 6: Posterior Parameter Estimates of Reward Prediction Error Floored Model. Shown is a pair plot of the bootstrapped (10,000 samples) posterior parameter estimates. The diagonal plots contains the histograms of the posterior parameter distribution of each parameter in the model. Importantly, the best (median value of the posterior) estimate of γ is ≈ 0. This means the Expected Value Floored Model converges to the Reward Valence Floored Model. The off diagonal plots show joint distributions for each pair of parameters (y-axis and x-axis). 95% confidence intervals are indicated with dark grey lines. Thick dark green lines correspond to the cumulative distribution.

Supplementary C: Error Corrections over Time in Experiment 1 and 2

In total, we had 48 clamps in Experiment 1 and 2 each that could either be a left, right, or center clamp (jittered on the target). As stated in the main text, these clamps were pseudorandomized to keep the subjects naive to the feedback manipulation, which protected their sense of contingency in the task. More specifically, these clamps were pseudorandomized into 6 “super-blocks” of 8 clamps. The blocked pseudorandomization helped ensure a more even distribution of types of error clamps throughout the experiment. This prevented problematic cases where many clamps in a row were all the same direction. The purpose here was again to protect participant contingency. Because of this super-blocking structure, despite the pseudorandomization, we can parse our error corrections across the experiment by collapsing the left and right clamp corrections across the 6 super-blocks (and multiplying our right clamp corrections by -1 to ensure all corrections are positive values). We ran a 2-way (Reinforcement Probability x Error Clamp Super-Block) Mixed ANOVA on these data in Experiment 1 and 2. We again found a main effect of reinforcement probability (“long-history” in main text) (F(1,38) = 7.35, p = 0.10, η^2 = 0.16), no main effect of error clamp super-block (F(5,190) = 2.09, p = 0.068, η^2 = 0.05), and no interaction (F(1,190) = .40, p = 0.85, η^2 = 0.01). The effect of reinforcement probability suggests that expected value does indeed modulate error corrections. Finding no main effect of error clamp super-block suggests that these differences manifest relatively early on and reach steady state within the first 8 error clamps. For Experiment 2, we again found no significant effects of reinforcement probability (F(1,38) = 0.61, p = 0.44, η^2 = 0.02), nor error clamp super-block (F(5,190) = 0.62, p = 0.69, η^2 =0.02), and no interaction (F(5,190) = 1.19, p = 0.32, η^2 =0.03).

In **Supplementary Figure 7** we visually see relatively stable performance across the groups over time, with the difference between groups already manifesting in the first and second block. Our Experiment 1 best-fit Expected Value Model shows a similar trend. These results, combined with the analyses in our main text, support that the steady state influence of expected value occurs around the first super-block or within the first 8 error clamps.

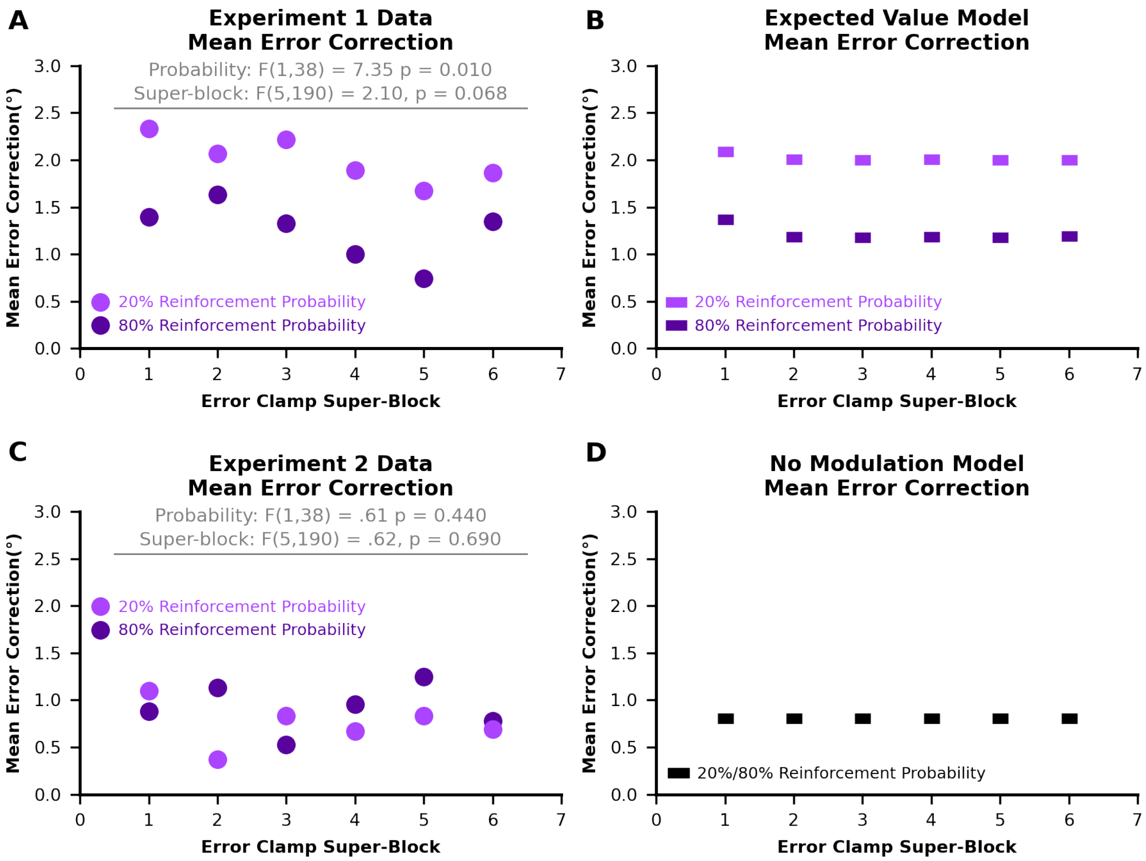

Supplementary Figure 7: Comparison between Experimental Data and Best-fit Expected Value Model. Plotted are the mean error corrections (y-axis) of experimental data and best-fit models for Experiment 1 and Experiment 2 collapsed across the pseudorandomized super-blocks through time (x-axis). **A)** We found no main effect of super-block over time, but again found a main effect of reinforcement probability. These results support that the steady state influence of expected value occurs at approximately the first super-block. **B)** Likewise, our best-fit Expected Value Model for Experiment 1 approaches steady state behaviour at approximately the first super-block. **C)** In Experiment 2 we found no main effect of super-block, and again no main effect of reinforcement probability. **D)** Likewise, our No Modulation model shows no effect of super-block.

Supplementary D: Influence of Response Time on Error Corrections

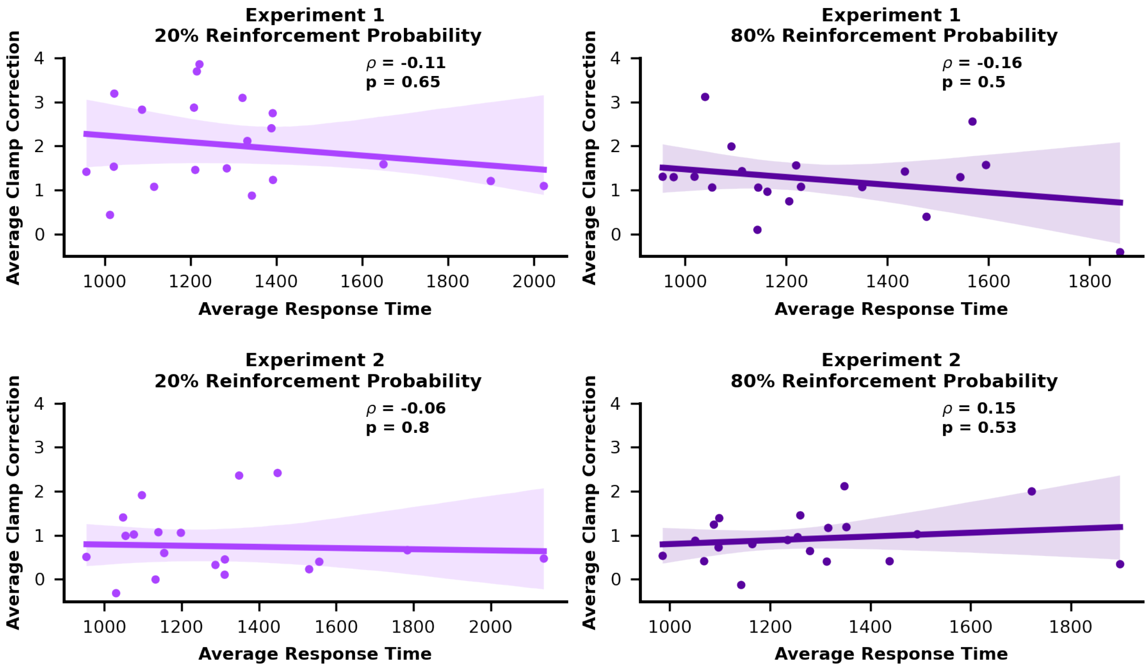

Supplementary Figure 8: Correlations (Pearson) Between Average Response Time and Average Correction. Plotted are the mean response time (x-axis) versus mean error corrections (y-axis) for each participant. Plotted in the upper right-hand corner are Pearson's ρ and p-value. We did not find any significant relationship between average response time and average clamp correction for either Experiment 1 ($p > 0.05$) or Experiment 2 ($p > 0.05$). All error clamps were collapsed across the short-history, and rightward clamp corrections were multiplied by -1 so all corrections were positive values. Plotted are best linear fits, and the shaded region shows the bootstrapped 95% confidence interval for the regression.
