# Supplementary material for: Successful reinforcement history suppresses explicit and implicit error corrections
Source: PLoS Comput Biol. 2026 Aug 3;22(8):e1014574. doi: 10.1371/journal.pcbi.1014574 (PMC13432137; doi:10.1371/journal.pcbi.1014574)
Supplement: Attachment — Submitted filename: ResponsetoReviewers.pdf [file pcbi.1014574.s003.pdf]

University of Delaware | College of Engineering
BIOMEDICAL ENGINEERING

May 18, 2026

Dear Dr. Blain and Dr. Marinazzo,

Thank you for the opportunity to resubmit our work. We would also like to thank the reviewers for their comments and suggestions, which have greatly improved the quality of this manuscript. We have addressed each of their comments and suggestions in a point-by point-manner, and added additional text and analyses to improve the clarity of the manuscript and methodology.

Below are the reviewer's comments (**bold and black text**) and our response (normal text). Changes to the manuscript are in bold and blue (**bold and orange text**).

Sincerely,

Joshua G. A. Cashaback on behalf of others

REVIEWER 1 COMMENTS

The authors present a study examining the influence of binary reinforcement feedback on explicit and implicit motor corrections made in response to clamped vector error feedback. Three behavioral experiments build on each other to show, collectively, that longer-term reinforcement history seems to preferentially impact explicit error corrections (experiments 1 and 2), while the immediate effect of reinforcement feedback signaling the absence of task error, despite visual feedback of a motor error, can reduce implicit corrections (experiment 3). In addition to behavioral results, the authors include an analysis using models of hypothesized potential mechanisms underlying the observed behavior. This model analysis shows that two candidate models can approximate the observed behavior. Specifically, a model that accounts for the expected value of actions can account for the modulation of explicit error corrections, while an error-based learning model that accounts for the presence of a task error can account for the modulation of implicit corrections. In both cases, the successful models outperformed alternative models/hypothesized mechanisms.

Overall, I found the paper interesting and think it adds to the body of literature attempting to understand the interaction(s) between reinforcement and supervised learning systems. Despite these strengths, I have some concerns/questions that I would like to see addressed before the manuscript is published. My specific comments are below.

We thank you for your interest, as well as the thorough and thoughtful review.

1. The mechanistic models and interpretation of the behavioral results in Experiments 1 and 2 make assumptions about the expected value of actions – i.e., that the actions of the 80% group would have a higher expected value than those of the 20% group. However, expected values are typically learned over time, and the analysis of error clamp trials appears to collapse all trials into a single subject mean. If I am correct, participants were not exposed to the probabilistic reinforcement during baseline, so it is not clear that participants would have formed an estimate of expected value until later in the task. Can the authors confirm whether performance was stable over time in their task, and, if so, discuss how this reconciles with typical theories of expected value?

As the reviewer correctly points out, the averaging of our point estimates across time ignores temporal information of expected value. We can parse the timescale by binning our error clamps. In total, we had 48 clamps in Experiment 1 and 2 each that could either be a left, right, or center clamp. As now stated in the main text, these clamps

were pseudorandomized into 6 "super-blocks" to ensure a more uniform distribution of types of error clamps throughout the experiment. This prevented problematic cases where many clamps in a row were all the same direction.

To test whether our differences between the 20% and 80% Reinforcement Probability group changed over these super-blocks, we ran a 2-way (Reinforcement Probability x Error Clamp Super-Block) Mixed ANOVA on these data in Experiment 1 and 2. We again found a main effect of reinforcement probability ("long-history" in main text) ($F(1,38) = 7.35$, $p = 0.010$, $\eta^2 = 0.16$), no main effect of error clamp super-block ($F(5,190) = 2.09$, $p = 0.068$, $\eta^2 = 0.05$), and no interaction ($F(1,190) = .40$, $p = 0.85$, $\eta^2 = 0.01$). As expected in Experiment 2, we again found no main effects of reinforcement probability ($F(1,38) = 0.61$, $p = 0.44$, $\eta^2 = 0.02$), nor a main effect of error clamp super-block ($F(5,190) = 0.62$, $p = 0.69$, $\eta^2 = 0.02$), and no interaction ($F(5,190) = 1.19$, $p = 0.32$, $\eta^2 = 0.03$). The effect of reinforcement probability in Experiment 1 suggests that expected value does indeed modulate explicit error corrections. Finding no main effect of error clamp super-block suggests that these differences manifest relatively early on and reach steady state within the first 8 error clamps.

Below, we plot the average error corrections for these super-blocks across time for our Experiment 1 and 2 data and our best-fit models.

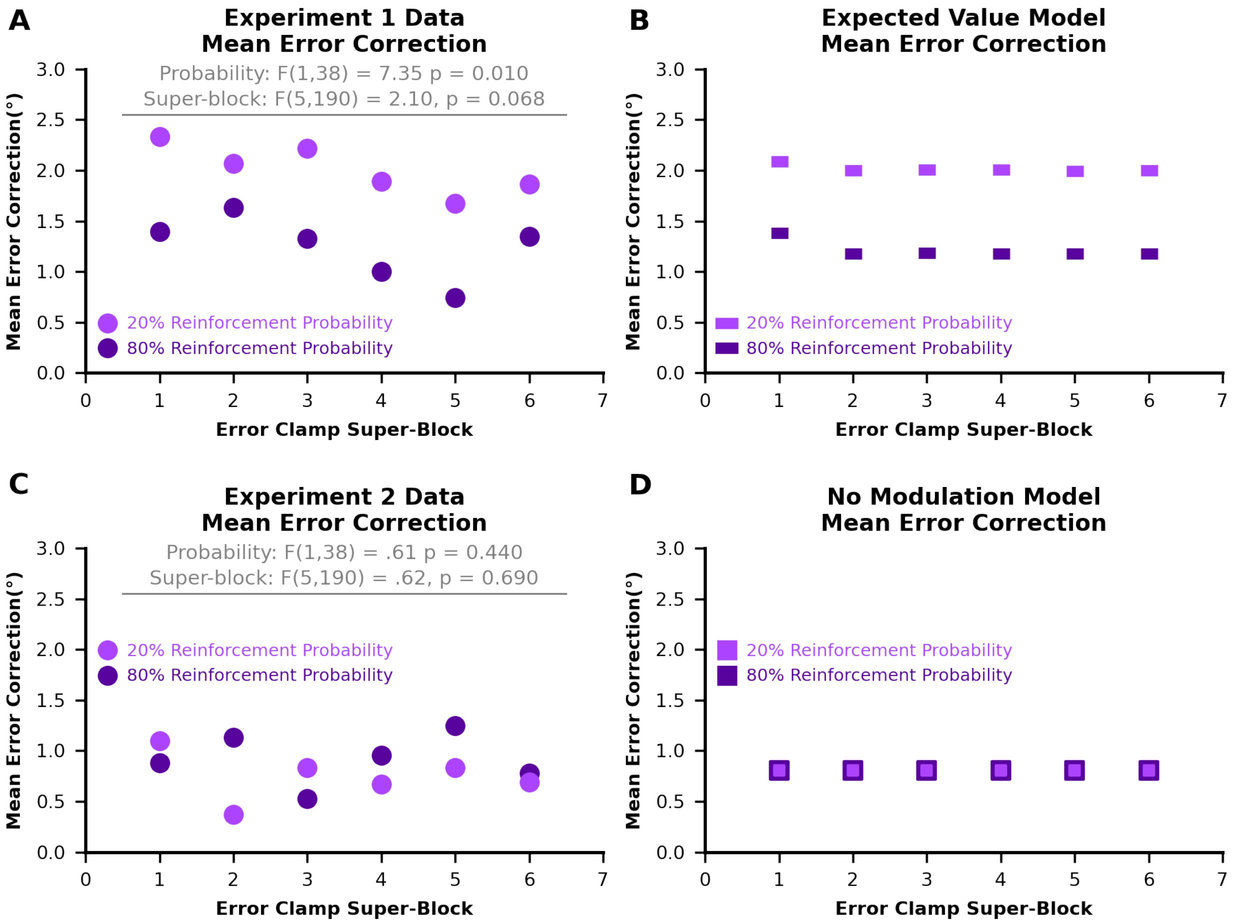

Supplementary Figure 7: Comparison between Experimental Data and Best-fit Expected Value Model. Plotted are the mean error corrections (y-axis) of experimental data and best-fit models for Experiment 1 and Experiment 2 collapsed across the pseudorandomized super-blocks through time (x-axis). **A)** We found no main effect of super-block over time, but again found a main effect of reinforcement probability. These results support that the steady state influence of expected value occurs at approximately the first super-block. **B)** Likewise, our best-fit Expected Value Model for Experiment 1 approaches steady state behaviour at approximately the first super-block. **C)** In Experiment 2 we found no main effect of super-block, and again no main effect of reinforcement probability. **D)** Likewise, our No Modulation model shows no effect of super-block.

Visually, we see relatively stable performance across the groups over time, with the difference between groups already manifesting in the first and second block. Our Experiment 1 best-fit Expected Value Model shows a similar trend. These results, combined with the analyses in our main text, support that the steady state influence of expected value occurs around the first super-block or within the first 8 error clamps.

We have included the text and figure above into **Supplementary C**, as well as adding the following text to the main manuscript.

Lines 601-605: **Our pseudorandomized procedure for error clamp types also contributed to participant naivety. We organized every 8 error clamps into a "super-block". In each super block we had 2 left clamps, 2 right clamps, and 4 clamps within the target center. Our pseudorandomization prevented the successive presentation of more than two of the same clamp type.**

Lines 176-179: **Given that expected value is a temporally evolving quantity, we examined how error corrections changed across the course of the entire experiment (see Supplementary C). We found evidence that expected value's modulatory effect on error corrections reached steady state within the first 8 error clamps.**

2. Relatedly, did all participants reliably reach within the reward region in Experiments 1 and 2? For example, were there any participants in the 80% group who missed the reward region on enough trials to have had an impact on the total reinforcement rate?

We thank the reviewer for this important question. The purpose of our reward region was simply to prevent the reinforcement of subject reaches that vastly exceeded their baseline variability. However, as expected our actual average reward rates are not equal to the experimentally defined rates, and the 80% reinforcement group deviated slightly more from the experimentally set reward rate. In Experiment 1 the 80% probability group's actual reward rate was 67.60% and the 20% probability group's actual reward rate was 18.35%. Importantly, despite the deviation from the "ideal" reinforcement rate the actual reinforcement rates are statistically different ($p < 0.001$). Similarly for Experiment 2, the actual reward rates were 68.70% for the 80% probability of reward group, and 19.11% for the 20% probability of reward group. The relative difference in reward rates in Experiment 2 was also significant ($p < 0.001$). Therefore, despite deviations from the experimentally set reward probabilities we were still effective in manipulating the relative difference in reward rates between the groups in Experiment 1 and Experiment 2.

We added additional text to emphasize that the actual reward rates were different between the groups:

Lines 164-166: **We confirmed that participants in the 20% and 80% reinforcement group had different actual reward rates (18.35% vs. 67.60%, $p < 0.001$).**

Lines 202-204: **We again confirmed that participants in the 20% and 80% reinforcement group had different actual reward rates (19.11% vs. 68.70%, $p < 0.001$).**

3. For all experiments, the authors report corrections in a single direction (i.e., more positive is larger), but the methods state that the direction of the error clamp

was counterbalanced across trials. Am I correct in assuming the authors flipped one of the error-clamp conditions? Given that they are measuring the response to vector error clamp feedback, it is important to ensure that corrections are made in the direction countering the imposed errors; otherwise, it is difficult to be sure that participants were responding to the error at all. Could the authors either clarify how their error corrections are reported or include an analysis of corrections relative to the error direction?

Yes, you are correct. Corrections to rightward clamps were multiplied by -1 to ensure when the clamps were collapsed across direction that all corrections had the same sign. We counterbalanced the direction to ensure that any directional bias would be mitigated. We have added additional text to our Experiment 1 and 2 Design section to highlight this:

Lines 609-612: **Error corrections to right error clamps were multiplied by -1 so that corrections to both right and left clamps were expressed as positive values. The mean error correction was taken across all error corrections as the main dependent measure for each participant.**

4. In Experiments 1 and 2, why was the baseline variability, which was used to set the width of the reward region, calculated using a mix of trials with and without visual feedback (20 and 20, if I am interpreting the methods correctly)?

Because participants experienced a mixture of reinforcement and visual feedback, we believed a reasonable way to calculate baseline variability was a composite of trials with no visual, and visual feedback. Our baseline block provided 50 trials total. The first 10 were practice, and we calculated baseline variability off of the next 20 with visual feedback, and the last 20 which had no visual feedback. We have added additional text to clarify this experimental design decision:

Lines 562-565: **The last 40 trials contained a mixture of visual (first 20 trials) and no visual feedback trials (last 20 trials). This mixture is representative of our task, given it was a mix of visual and no visual feedback.**

5. Were the short history of reward clamp conditions in Experiments 1 and 2 counterbalanced across trials within each reinforcement group? Or did each group get a fixed short-history condition?

We thank the reviewer for noticing our omission of this experimental detail. The short history of reward clamps were pseudorandomized. 50% of the reinforcement clamps were a successful short history (reinforcement, reinforcement) and the other 50% of the clamps were a less successful short-history (reinforcement, no reinforcement). The order

of these clamps were pseudorandomized within each 8 error clamp super-block. We have added these details to the manuscript:

Lines 576-580: **Half of the short-history clamps were a successful short-history and half were a less successful short-history. The order of these clamps were pseudo-randomized within "super-blocks" of 8 clamps. Our pseudorandomization prevented the successive presentation of too many of the same short-history clamp type.**

6. For Experiment 3, can the authors explain the choice to provide reinforcement feedback whenever participants hit the target in the larger+reinforcement condition, but only provide punishment when participants were in the reward region in the small+punishment condition? How frequently did participants miss the target by reaching outside the reward region in the small+punishment condition? I am concerned that inconsistent punishment feedback could have led participants to discount or ignore it, which could explain the lack of modulation observed in that condition (rather than it resulting from differential processing of reward versus punishment).

Thank you for catching this error within our text. No reinforcement region was necessary in Experiment 3, as participants always had visual error feedback. Punishment feedback was therefore ***not*** contingent upon reaching within a reinforcement region (this also would make no sense). If participants missed the target during a punishment trial, they received punishment. We have fixed this in our manuscript:

Lines 626-627: **In the small target punishment condition, participants received punishment when they missed the target.**

7. It is not totally clear how the authors think the results and top-performing models from the three experiments presented here go together. In Experiments 1 and 2, the short-term history of reward is nested within the long-term history. That is, to have a reward prediction error, the system must have some knowledge of the reward history. In Experiment 3, however, the immediate history of reward is treated as something independent of any representation of the reward history. Could the authors clarify their thinking about this in the discussion, or when justifying the design of Experiment 3?

The results of Experiment 1 and 2 suggest that the modulation of error corrections by reinforcement history is dominated by explicit mechanisms. However, previous studies have shown that removing task errors (or providing success) on the same trial as an error (e.g. Leow et. al (2018), Kim et. al (2019), Al-Fawakhiri et. al (2023)) attenuates implicit adaptation. Experiment 3 was performed to investigate this issue, as well as round out

our line of questioning. Experiment 1 and 2 did not provide reinforcement on the same trial as an error clamp to intentionally focus on historical effects, so Experiment 3 was run to fill in this gap.

In Experiment 3, we found reinforcement had an attenuating effect on implicit error corrections when presented on the same trial as an error. Similar to Al-Fawakhiri and colleagues (2023), we were unable to replicate the effect of target size ($p = 0.19$) on implicit error corrections seen in Kim et. al (2019). Again, similar to Al-Fawakhiri and colleagues, the visual trends in our data were still what you would expect from the elimination of a task error. Our reinforcement feedback (visual and auditory stimuli, and money) and within-subjects design was chosen to maximize any effect sizes. Because we did find an immediate influence of reinforcement on implicit corrections, these inconsistencies led us to hypothesize that perhaps a single reward based mechanism explains both our findings and the effects of target size on implicit error corrections. This could explain the unreliable effects in the literature, and the reinforcement feedback we provided may merely have been increasing the "gain" on the same mechanism that drives target size effects.

Cumulatively, our three experiments suggest that modulation of implicit error corrections by reinforcing stimuli does not really rely on history at all. Because this mechanism occurs immediately before an error correction we decided to call its timescale the "immediate history". We attempt to explain this mechanism through our reward valence model. As discussed in Supplementary B, this is equivalent to a reward prediction model or expected value model that only considers immediate rewards, or the current value of the reward. This model assumes an implicit error correction is modulated by the magnitude of the reinforcing stimuli itself.

The general findings would then suggest a higher-level cognitive representation of expected value that influences explicit error corrections, and a lower-level implicit process influencing implicit error corrections. To better justify Experiment 3 and to clarify our interpretation of our findings we have added the additional text:

Lines 239-242: **Furthermore, previous literature shows inconsistent effects of reinforcing stimuli given on the same trial as a visual error (Leow et. al (2018), Kim et. al (2019), Al-Fawakhiri (2023)), which we address with a relatively larger sample size and within subjects design.**

Lines 397-398: **Therefore, our extrinsic reinforcement feedback may simply be a gain on a shared mechanism. Targets hits may generate an "intrinsic" reinforcement signal.**

Lines 422-425: **Together, these models suggest that the reinforcement history in-**

fluences error corrections through a higher-level explicit representation of expected value, and that a reinforcing stimuli during an error effects implicit error corrections through a lower-level mechanism acting through the reinforcer.

8. The authors' interpretation of their results seems to treat explicit and implicit error corrections as non-overlapping, but it is likely that both correction processes operate together to drive behavior in daily life. I am wondering whether the authors could discuss the ways their results elucidate the interaction between explicit and implicit corrections. I think this issue is particularly relevant when interpreting the result of Experiment 3, which included a manipulation of task errors. The authors point out that task errors are considered by some to be reinforcement signals, but task errors are also thought to be a significant driver of explicit corrections (and that the link to reinforcement learning is via explicit mechanisms).

We agree with the reviewer that explicit and implicit processes overlap. Unfortunately, our results cannot directly unpack the interaction between explicit and implicit processes, as we experimentally constrain explicit corrections in Experiment 3. Future work could seek to manipulate explicit corrections directly to elucidate this topic.

Our results are fully compatible and consistent with previous work that emphasizes how reinforcement and explicit task errors (distance of the end effector from a target) influence explicit action selection (e.g. Mazzoni and Krakauer (2006), Shadmehr et. al (2011), Taylor and Ivry (2011), Holland et. al (2018)). Our results are fully consistent with an explicit component to task errors. Our concern is the interpretation of the *implicit* effects of task errors. We have shown there is a qualitative distinction between the way the explicit and implicit systems utilize reinforcement stimuli. And, given that the implicit effect of reinforcement feedback is similar to the implicit effect of task errors in the literature, we suggest that these two phenomena may be needlessly differentiated and could be the same mechanism.

We have added the additional text to clarify our thinking. We have also changed the terminology in our discussion to specify "implicit task errors" when relevant. We have also added text to suggest how future work could address the interactions between explicit and implicit corrections, potentially by directly manipulating explicit strategies.

Lines 330-332: **This suggests the utilization of expected value by the explicit system is a higher-level cognitive process and that same quantity is not influencing implicit error corrections.**

Lines 379-381: **Task errors are known to drive explicit action selection (Mazzoni and Krakauer (2006), Shadmehr et. al (2011), Taylor and Ivry (2011), Holland et. al (2018)). We controlled for explicit corrections in Experiment 3, and here were**

primarily interested in the *implicit* effects of task error.

Lines 447-449: **Future work could address the interactions between explicit and implicit corrections, potentially by directly manipulating explicit strategies.**

9. I noticed a few typographical errors:
Page 2, line 55, should be modulates
Page 14, line 242, either "corrected" or "to" is incorrect here
Page 26, line 531, missing "subjects"

Thank you. These have been corrected.

Line 55-56: **One possibility is that expected value modulates error corrections, which would optimize average success.**

Lines 269-271: **Fig. 4B shows a representative participant illustrating how the feedback conditions were pseudorandomized and how participants responded to error clamps.**

Line 620: **We used a within subjects design where participants experienced one of four possible conditions: large target, large target with reinforcement, small target, and small target with punishment (Fig. 4A).**

REVIEWER 2 COMMENTS

Reviewer 2: In the article "Successful reinforcement history suppresses explicit and implicit error corrections," the authors look to determine the impact of reinforcement history on explicit (cognitive) and implicit (involuntary) motor corrections. Three experiments were conducted in which the authors compared two competing hypotheses: (1) reward prediction errors (surprise) modulate explicit and implicit error corrections and (2) expected value (average reward) modulates explicit and implicit error corrections. Results, experimental behaviour and computational modeling, suggest that expected value modulates explicit error corrections. An immediate history of reinforcement modulates implicit error corrections. Together, results indicate that a successful reinforcement history suppress explicit and implicit error corrections. The authors ask an interesting question, and I appreciate the clever methodology adapted to manipulate reward prediction errors and expected value. Below I outline several questions, mainly dealing with clarification around the methodology.

We thank the reviewer for their interest in our question, thoughtful comments, and pos-

itive remarks on our methodology.

The word reinforcement is used to refer to movement outcome (success or failure), as well as additional extrinsic feedback. How do these reinforcement sources interact?

We view both movement outcome (intrinsic) and extrinsic feedback as reinforcers, which is consistent with classic theories. Schultz and colleagues (2006) state that rewards are not necessarily an objective physical property of a stimulus, but are defined by their internal reward function (Schultz et. al (2006)).

We provide extrinsic, binary ("on" or "off") reinforcement feedback that present three kinds of stimuli simultaneously, i.e. pleasant auditory stimuli, visual stimulus, monetary reward. Past research shows these three kinds stimuli to be reinforcing (Blatter & Schultz (2005), Ferrerri et. al (2019), Delgado et. al (2003)). Binary reinforcement feedback is an effective stimulus that results in human subjects learning to select an action (such as a particular reach) that maximizes their reinforcement feedback (e.g. Shadmehr et. al (2011), Huang et. al (2011), Therrien et. al (2018), Roth (2023)).

Achieving movement goals has been shown to be intrinsically rewarding and involves the dopamine system. For example, dopaminergic neurons in the ventral tegmental area (VTA) of zebra finches respond to hitting or missing sensorimotor targets during song practice, supporting the existence of an intrinsic reinforcement signal during motor learning (Gadagkar et. al (2016)).

In Experiment 1 and 2 extrinsic and intrinsic reinforcers are not present simultaneously. However, in Experiment 3 we have both a Large Target condition, where subjects are shown manipulated cursor feedback inside a target, and a Large Target Reinforcement condition, where subjects are again shown a cursor embedded in a large target but receive additional extrinsic reinforcement feedback. Although we did not replicate the finding of Kim et. al (2019) that accomplishing a motor goal attenuates implicit error corrections (p=0.18), the Large Target condition was trending in the expected direction. This is relatively unsurprising, as we used single trial probes whereas Kim and colleagues had participants adapt continuously to a rotation. Kim and colleagues did not find a statistical difference in their early adaptation rate in the Experiment 1, and the effect of target size was only shown after many trials. However, we did find that external reinforcement feedback attenuated implicit error corrections in Experiment 3 (p=0.016). This would suggest that the effects of our external reinforcement feedback were additive in nature to any intrinsic reinforcement provided by task success.

We have clarified our definitions and thinking about reinforcers, and their contribution to our task in the following text:

Lines 508-509: **When participants were given extrinsic reinforcement feedback the target briefly turned blue and expanded...**

Lines 394-396: **Therefore, our extrinsic reinforcement feedback may simply be a gain on a shared mechanism. Targets hits may generate an "intrinsic" reinforcement signal.**

Line 511: **Past research shows visual, auditory, and monetary stimuli to be reinforcing (Blatter & Schultz (2005), Ferrerri et. al (2019), Delgado et. al (2003)).**

1. Do participants see cursor feedback throughout the reach? If not, how does this influence sensory prediction errors and underlying implicit corrections?

To make sure participants did not move their hand outside the home position before the start of each trial, they had continuous cursor feedback when within 1.6 cm of the home location. That cursor feedback disappeared after the go cue.

All other cursor feedback in the experiment was only endpoint feedback. Endpoint feedback prevents continuous feedback corrections during reaching and reaching at self selected movement speeds, without the need to constrain to fast movement speeds.

During a reach to the target, participants only have endpoint cursor feedback when it is provided. In Experiment 1 and 2 this occurs during baseline and error clamps. In Experiment 3 participants received endpoint cursor feedback on every reach after baseline. Endpoint cursor feedback is seen immediately when participants pass through the target, and there is no delay. This is important, as delayed feedback has been shown to eliminate implicit error corrections (Shween and Hegele (2017)). Previous work shows that continuous error feedback results in stronger implicit error corrections (Hinder et. al (2008), Taylor et. al (2014)). However, implicit error corrections still proceed with endpoint feedback (Mazzoni and Krakauer (2006), Hinder et. al (2008), Taylor et. al (2011), Taylor et. al (2014), D'Amario et. al (2024)). For one, endpoint feedback prevents continuous feedback corrections during participant reaches. We were interested in endpoint corrections and ensured our error corrections were a measure of adjusted feedforward motor commands. Also, in our task endpoint feedback was important to protect contingency. Contingency is an important aspect of reinforcement (Schultz et. al (2006)). Clamped continuous feedback would likely have made participants suspicious as their feedback would've been fixed along a trajectory. We wanted to ensure participants' reinforcement felt contingent on their behaviour and felt free to make explicit adjustments.

We have added additional text in our methods section to justify our choice in using endpoint feedback:

Lines 516-519: **Endpoint feedback has been used effectively to induce implicit and**

explicit error corrections (Mazzoni and Krakauer (2006), Hinder et. al (2008), Taylor et. al (2011), Taylor et. al (2014)). Endpoint feedback also prevents feedback error corrections, which ensures our metrics were measuring feedforward motor commands.

2. Participants were to move through the target. If they reached through a reward zone, they then had a 20% or 80% chance of receiving feedback on each trial in Experiments 1 and 2. Did this ensure that participants received feedback on 2 and 8 of the 10 initial trials?

In Experiment 1 and Experiment 2 participants performed 48 blocks of 13 trials. The first ten trials of each of those blocks provided probabilistic reinforcement feedback on each trial. For each trial, that probability of receiving reinforcement (20% or 80%) that depended on participant group. Also, this probabilistic feedback was conditioned on whether participants reached through a reward region that was scaled by their baseline variability. This was to prevent reaches from being rewarded that were quite far from the target. The rationale of this approach was to protect participant contingency. Because of participant random walk behaviour (Roth et. al (2023)), and the fundamentally probabilistic nature of the feedback participants in each group the actual reward rate of each group was not 2 or 8 out of the 10 initial trials.
We have added additional text to clarify our methods, and the actual reward rates participants received:

Lines 548-550: **A consequence of this probabilistic methodology is that any individual would not necessarily receive an actual reward rate of 20% or 80%.**

Lines 165-167: **We confirmed that participants in the 20% and 80% reinforcement group had different actual reward rates (18.35% vs. 67.60%, p <0.001).**

Lines 206-208: **We again confirmed that participants in the 20% and 80% reinforcement group had different actual reward rates (19.11% vs. 68.70%, p <0.001).**

3. In the error clamp trials (t-3 and t-2), did the cursor feedback go through the target? What happened on trial (t-2) if reinforcement feedback was not provided?

The [t-3] and [t-2] trials in the blocked design of Experiment 1 and 2 only provided binary reinforcement feedback. If participants did not receive reinforcement on the [t-2] trial no feedback was provided to the participant on their performance.

We have clarified our methodology with the additional text and update to our figure caption:

Fig 1. caption: **No endpoint feedback was given on short-history reinforcement**

clamps.

Lines 575-576: **Participants received only binary reinforcement feedback and no endpoint feedback on short-history reinforcement clamps.**

In Figure caption 1 it indicates that reinforcement feedback was not contingent on performance. Is this the case? Previous text indicated that feedback was only provided if reaches were through a reward region.

We have updated our text to clarify that reinforcement feedback was contingent on hitting the visual target:

Fig 1 caption: **Unbeknownst to participants, the reinforcement feedback was probabilistic and was not contingent on hitting the visual target.**

5. Can the reinforcement and punishment feedback be considered equivalent? Is the reinforcement feedback processed as rewarding as the punishment feedback is processed as punishing?

We thank the reviewer for this question about verifying our reinforcement and punishment stimuli. Our reinforcement feedback, similar to our punishment feedback, was multimodal. We provided a visual (blue target vs. red), auditory (pleasant noise vs. angry buzzer), and monetary (increase or decrease in performance bonus) stimulus. Therefore the stimuli were physically different. Secondly, in Experiment 3 participants completed 7-point Likert scales to verify that reinforcement and punishment feedback were interpreted correctly and oppositely. They responding to the statements, "It felt good when I hit the target and it turned blue" and "It felt bad when I missed the target and it turned red" after completing the experiment. On average participants agreed with both statements (average score for reward: 6.58, average score for punishment 6.03). Finally, given that our reinforcement feedback resulted in attenuated error corrections and our punishment feedback did not, this suggests our feedback was processed differently.

We have now included our Likert score data for Experiment 3 in our methods to provide verification of our reinforcement and punishment stimuli:

Lines 627-632: **Participants completed 7-point Likert scales to verify that reinforcement and punishment feedback were interpreted correctly. They responded to the statements, "It felt good when I hit the target and it turned blue" and "It felt bad when I missed the target and it turned red". On average participants agreed with both statements (average score for reward: 6.58, average score for punishment: 6.03).**

6. Was the target shown before the go signal? If so, how did this long preparation time influence the engagement of explicit/implicit processes?

The reaching target was always present during the experiment. Participants waited between 250 to 1000ms (uniformly drawn) for a go cue after entering the target. Participants could not initiate a reach before the go cue. Greater preparation time is known to reduce anticipatory behaviour (Tsay et. al (2022)). By randomly drawing preparation time from a uniform distribution our aim was to prevent participants from anticipating the go cue, while randomizing it as an experimental variable to minimize any systematic effects. This facilitates active engagement in the task. Participants were verbally encouraged to initiate a reach at the presentation of the go cue, but we did not force a particular response time. Therefore, in our experiments there was no direct manipulation of preparation time.

However, we are able to look at the self-selected *response time* and see its influence on the explicit and implicit process. In Experiment 1 we allowed participants to make both explicit and implicit corrections. In Experiment 2 we controlled for explicit corrections and participants were only free to make implicit corrections. Both experiments were otherwise identical. Given we had the exact same methodology across both, we were able to parse how the explicit/implicit processes could be affected by self-selected preparation time.

We pooled subject error corrections across group both in Experiment 1 and Experiment 2 in **Supplementary D**. We then calculated participant response times on each trial they performed an error correction. However, we did not find any evidence that self-selected preparation time correlated with error correction magnitude in Experiment 1 (20%: $\rho = -0.11, p = 0.65$, 80%: $\rho = -0.16, p = 0.5$) or Experiment 2 (20%: $\rho = -0.06, p = 0.8$, 80%: $\rho = 0.07, p = 0.78$).

We have included these analyses in our Supplementary Material:

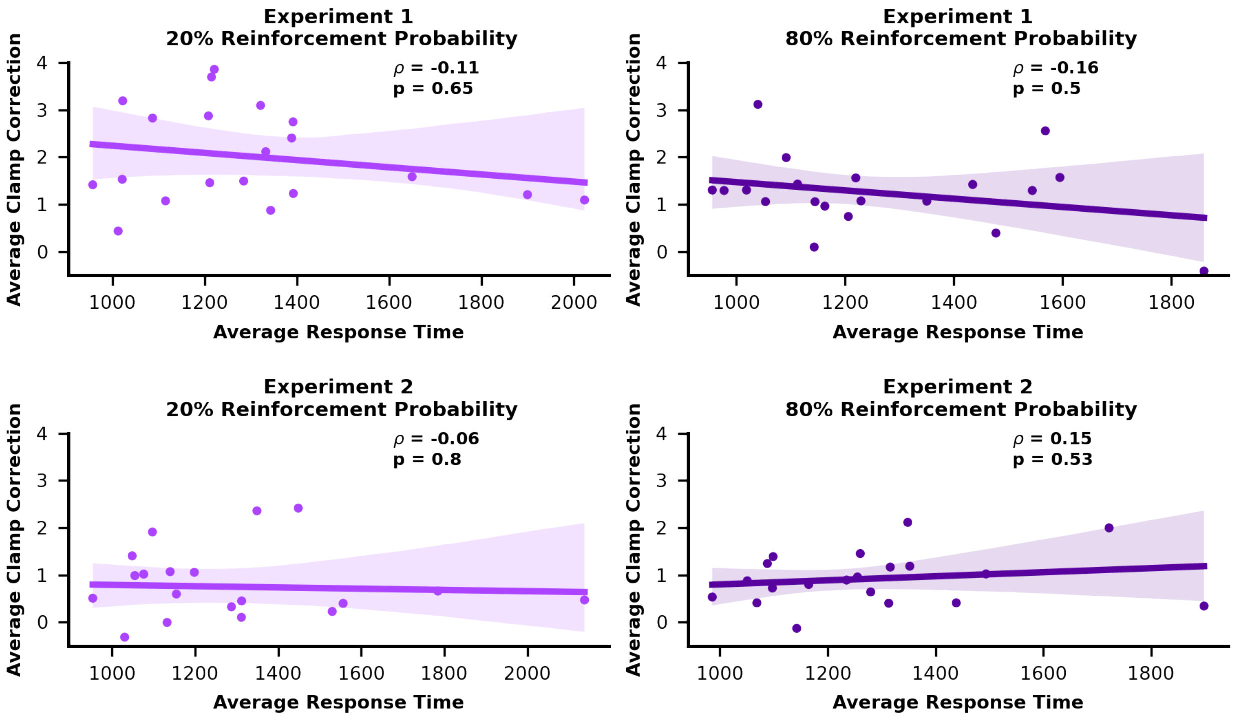

Supplementary Figure 8: Correlations (Pearson) Between Average Response Time and Average Correction. Plotted are the mean response time (x-axis) versus mean error corrections (y-axis) for each participant. Plotted in the upper right-hand corner are Pearson's ρ and p-value. We did not find any significant relationship between average response time and average clamp correction for either Experiment 1 ($p > 0.05$) or Experiment 2 ($p > 0.05$). All error clamps were collapsed across the short-history, and rightward clamp corrections were multiplied by -1 so all corrections were positive values. Plotted are best linear fits, and the shaded region shows the bootstrapped 95% confidence interval for the regression.

We have also added the additional text to clarify our methods, and highlight the purpose and consequences of our randomized go cue:

Lines 496-498: **Participants were verbally encouraged to initiate a reach at the presentation of the go cue.**

Lines 501-506: **The purpose of this procedure was to prevent anticipatory, erroneous reaches. In Supplementary D, we analyze average response times of participants and show there was no influence of response time on mean error corrections for Experiment 1 and 2. That analysis supports response time did not effect the relative engagement of the explicit versus implicit process.**

7. Was there only 1 target?

Yes, there was only one target. We clarified this in the text:

Line 86 **...a single circular target...**

Line 487: **In all experiments, participants saw a single, circular target.**

How does use-dependent error corrections/learning factor into the current results?

In upper extremity tasks use-dependent learning is typically measured after participants repeat movements towards a particular target, and then a bias is measured towards that target relative to another (Diedrichsen et. al (2010), Verstynen and Sabes (2011), Tsay et. al (2022)). In our experiments, participants made reaches towards a single target. Therefore, unless participants were reaching on average to different locations for different conditions, we would not expect use-dependent learning to affect the relative differences between our conditions in this study. We confirmed that average reach angle was not different between the 20% and the 80% condition in Experiment 1 (p = 0.29) and Experiment 2 (p=0.68). Experiment 3 was a within-subjects experiment, and conditions were pseudorandomized uniformly in blocks and error clamps were counterbalanced by direction throughout the experiment. Thus any use-dependent learning should be constant across conditions. Furthermore, if anything, use-dependent learning would be negatively effecting our statistical power. Use-dependent learning causes future movements to resemble past movements and thus would be working against any error correction.

Is the factor of the short-history of reinforcement clamps factored into the results (RC1 versus RC2)?

Yes. We did not find a main effect the short-history of reinforcement on our results ($F(1,38) = 3.51$, $p = 0.069$, $\eta^2 = 0.08$). Therefore, in our results **Figure 3** the mean error corrections show our data collapsed across the main effect of short-history.

We have clarified this by adjusting our figure caption:

Figure 3 caption: **Mean error corrections are collapsed across short-history clamps, as there was no main effect of short-history.**

Was the factor of block (i.e., time) considered in the current results?

Reviewer 1 asked a similar question. For you convenience we have copied the response below:

As the reviewer correctly points out, the averaging of our point estimates across time ignores temporal information of expected value. We can parse the timescale by binning our error clamps. In total, we had 48 clamps in Experiment 1 and 2 each that could

either be a left, right, or center clamp.As now stated in the main text, these clamps were pseudorandomized into 6 “super-blocks” to ensure a more uniform distribution of types of error clamps throughout the experiment. This prevented problematic cases where many clamps in a row were all the same direction.

To test whether our differences between the 20% and 80% Reinforcement Probability group changed over these super-blocks, we ran a 2-way (Reinforcement Probability x Error Clamp Super-Block) Mixed ANOVA on these data in Experiment 1 and 2. We again found a main effect of reinforcement probability (“long-history” in main text) (F(1,38) = 7.35, p = 0.010, η^2 = 0.16), no main effect of error clamp super-block (F(5,190) = 2.09, p = 0.068, η^2 = 0.05), and no interaction (F(1,190) = .40, p = 0.85, η^2 = 0.01). As expected in Experiment 2, we again found no main effects of reinforcement probability (F(1,38) = 0.61, p = 0.44, η^2 = 0.02), nor a main effect of error clamp super-block (F(5,190) = 0.62, p = 0.69, η^2 =0.02), and no interaction (F(5,190) = 1.19, p = 0.32, η^2 =0.03). The effect of reinforcement probability in Experiment 1 suggests that expected value does indeed modulate explicit error corrections. Finding no main effect of error clamp super-block suggests that these differences manifest relatively early on and reach steady state within the first 8 error clamps.

Below, we plot the average error corrections for these super-blocks across time for our Experiment 1 and 2 data and our best-fit models.

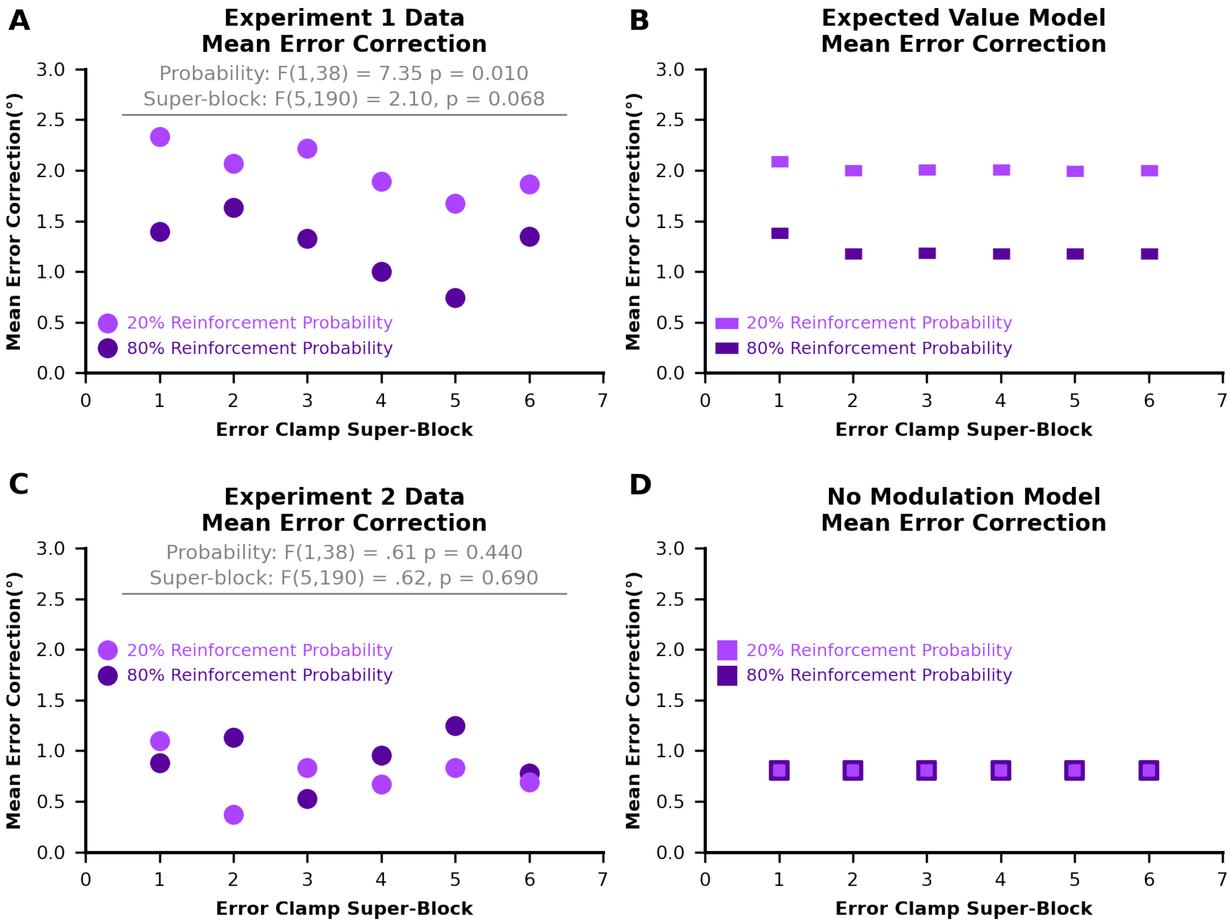

Supplementary Figure 7: Comparison between Experimental Data and Best-fit Expected Value Model. Plotted are the mean error corrections (y-axis) of experimental data and best-fit models for Experiment 1 and Experiment 2 collapsed across the pseudorandomized super-blocks through time (x-axis). **A)** We found no main effect of super-block over time, but again found a main effect of reinforcement probability. These results support that the steady state influence of expected value occurs at approximately the first super-block. **B)** Likewise, our best-fit Expected Value Model for Experiment 1 approaches steady state behaviour at approximately the first super-block. **C)** In Experiment 2 we found no main effect of super-block, and again no main effect of reinforcement probability. **D)** Likewise, our No Modulation model shows no effect of super-block.

Visually, we see relatively stable performance across the groups over time, with the difference between groups already manifesting in the first and second block. Our Experiment 1 best-fit Expected Value Model shows a similar trend. These results, combined with the analyses in our main text, support that the steady state influence of expected value occurs around the first super-block or within the first 8 error clamps.

We have included the text and Figure above into **Supplementary C**, as well as adding the following text to the main manuscript.

Lines 603-607: **Our pseudorandomized procedure for error clamp types also contributed to participant naivety. We organized every 8 error clamps into a "super-block". In each super block we had 2 left clamps, 2 right clamps, and 4 clamps within the target center. Our pseudorandomization prevented the successive presentation of more than two of the same clamp type.**

Lines 172-175: **Given that expected value is a temporally evolving quantity, we examined how error corrections changed across the course of the entire experiment (see Supplementary C). We found evidence that expected value's modulatory effect on error corrections reached steady state within the first 8 error clamps.**

Previous research has shown early adjustments in reaches are typically driven by explicit processes, with implicit contributions increasing over trials.

Previous work has shown that in response to a constant perturbation an explicit correction occurs quickly, while it takes more time for the implicit component to compensate for the error (Taylor et. al (2014), McDougle et. al (2015), Bond and Taylor (2015)). Previous work also shows that explicit error corrections are better able to compensate for large (> 15°) visual perturbations (Bond and Taylor (2015)). This means for large, constant corrections the explicit component dominates. However, this does not mean the implicit system is not active early on. In fact, while implicit corrections accumulate during constant perturbations over time, have a maximum correction correction size early on, give they follow an exponential learning rule (Burge et. al (2008), McDougle et. al (2015), Zhang et. al (2024)). Furthermore, implicit error corrections have also been shown to be sensitive to smaller errors (Kim et. al (2018)), with the strength of early adaptation reaching a plateau at ≈ 5 degrees.

In our experiments the imposed error clamps were quite small. Clamps were 4.4° for Experiment 1 and 2 and 2° for Experiment 2. As shown in our main text, our corrections to these errors are also quite small. Small error corrections would help temper any advantage in correction size the explicit process would have over the implicit process. Also, small error corrections are less noticeable (Werner et. al (2015)) and awareness is related to more explicit involvement (Neville and Cressman (2018)). Both of these facts meant using a smaller clamp size should have resulted in us having comparable strengths of the explicit and implicit processes in our corrections. In confirmation, our average correction size for the explicit and implicit was approximately 2° for our 20% reinforcement probability group in Experiment 1. In Experiment 2, our measured implicit error corrections were slightly less than 1°. Simple subtraction reveals that the additional explicit component contributing to Experiment 1 was on the same order of magnitude (≈ 1°) as our isolated implicit corrections in Experiment 2.

We have clarified in our Methods how using small error clamps allows for a better relative

mixture of the explicit and implicit processes:

Lines 586-592: **Error clamps were relatively small for a few reasons. One was to protect participants' sense of contingency. Larger errors are more noticeable (Werner et. al (2015)). Secondly, we wanted to limit any potentially dominating effects of explicit corrections. Explicit error corrections compensate with greater magnitude and more quickly to large perturbations (McDougle et. al (2015), Bond and Taylor (2015)). Also, implicit corrections peak at error sensitivity at smaller error sizes (Kim et. al (2018)). Therefore, smaller clamp sizes ensured a more equal contribution of both processes.**

Conclusions drawn (e.g., lines 204-206; 289-291), assume that explicit and implicit corrections are independent. Research has shown that there are multiple explicit and implicit processes, and that these processes may interact (see Weightman et al., 2022; Larssen and Hodges, 2023)

As the reviewer rightly points out, there is strong evidence for an interplay, and perhaps an interaction, between the explicit and implicit component of error corrections. Two strong examples of this are incomplete additivity and the effect of task errors on implicit adaptation. Additivity is the concept that independent measurements of a component process sum linearly. In the case of visual, upper extremity tasks this would mean the total error correction would be the addition between the measured explicit and implicit components. This assumption is a simplifying one. Work by 't Hart and colleagues (2024) and Chen and Taylor (2026) suggest that only partial additivity is true and that independent *measurements* of explicit and implicit adaptation do not add perfectly to total adaptation. In regards to task errors, evidence suggests task errors (effector-to-target) modulate implicit adaptation (Leow (2018), Kim et. al (2019), Tsay (2022), Al-fawakhiri (2023)). Also, as the reviewer mentions, Larssen and Hodges (2023) show that augmented numerical feedback can affect implicit corrections. Task errors are dependent on explicit aim and therefore imply a coupling between explicit strategy and the degree of implicit adaptation. Both of these lines of evidence suggest an interplay or interaction between these two processes. In adaptation tasks, it is not sufficient to merely measure one and assume the other.

However, the logic of our conclusion across Experiment 1 and 2 does not rely on strict additivity of measurements nor independence. It relies merely on the exclusion of explicit corrections. Corrections are a composite of an explicit correction, an implicit correction, and any interaction between them. By preventing explicit corrections in Experiment 2, we isolate implicit error corrections from the explicit correction and any interaction. In Experiment 2, we did not find any direct influence of reinforcement history on the implicit process. This means that the effect we see in Experiment 1 must be a consequence of explicit error corrections, and/or the effect the explicit process has on the implicit pro-

cess (interaction).

We have clarified the limitations of assumptions of independence in our model, specified our language regarding our conclusions in lines 225-232 and 321-327, and suggested that future work could parse potential interactions:

Lines 717-718: **For simplicity, we assume no complex interactions between the processes (Albert et. al (2022)).**

Lines 225-232: **Error corrections are composed of an explicit correction, an implicit correction, and any explicit-implicit interaction. In Experiment 2, we eliminated explicit corrections and therefore isolated implicit error corrections from either the explicit correction and any explicit-implicit interaction. We did not find any direct influence of the long-history or short-history of reinforcement on implicit corrections. Critically, this means the influence of the long-history of reinforcement in Experiment 1 must be a consequence of explicit error corrections.**

Lines 450-452: **Future work could address the interactions between explicit and implicit corrections, potentially by directly manipulating explicit strategies.**

Minor

We thank the reviewer for carefully reading the text and providing lower level comments to improve readability.

Line 2: Does a basketball player expect to make many shots and is a miss surprising? Average shooting percentage, even for professionals, is less than 50%, so technically players miss more shots than they make.

We decided to keep our original wording, the original phrasing does not specify a particular percentage.

Line 64: missing a word; " ..., but could have been through ..."

We have updated the wording.

Line 64-66: **Our empirical results and computational modelling support that expected value modulates error corrections. Yet, Experiment 1 does not parse whether that effect is through explicit and/or implicit processes.**

Line 74: please clarify reward valence.

We have defined reward valence more precisely throughout our text.

Line 75-76: **...directly through reward valence, the signed reward magnitude.**

Line 123-124: **...reward valence (α), the signed reward magnitude.**

Line 286: **...α is the reward valence, the signed reward magnitude, and...**

Figure caption 1: missing the word 'to'; "from the 13th to 4th trial prior to an error correction ..."

Thank you. We have added the missing "t".

Figure caption 1: **...from the 13th to 4th trial prior to an error correction...**

Figure caption 1: the phrase 'participants were shown an error clamp (t-1) is unclear. Did participants not experience an error clamp trial as they reached?

Thank you. Participants did see an error clamp as their hand passed through the target. We have clarified our language:

Figure caption 1: **Following the short-history reinforcement clamps, on the next trial [t-1] participants experienced an error clamp as their hand passed through the target.**

Line 210: the phrase "immediate history that could be provided on the same trial as the error clamp" is confusing. Are you referring to the extrinsic reinforcement/punishment feedback being provided on trial (t-1) with the error clamp?

Thank you. Yes, in our first experiment we did not provided reinforcement feedback on our miss error clamps. We have clarified this sentence:

Lines 235-237: **However, this does not rule out the influence of an immediate-history. In Experiment 2, we did not provide reinforcement on the same trial as a miss error clamp (i.e. on the [t-1] trial).**

Line 357: missing the word 'a'; "... but they did find a difference ..."

We have updated out text.

Line 359: **...but they did find a difference in learning retention.**

Line 321: How gradients of feedback?

We have clarified out wording.

Line 360-361: **How graded reinforcement feedback interacts with the modulatory effects of reinforcement history is an interesting and open question.**

Line 478: How were participants educated on explicit re-aiming strategies? What were they told re the error clamp trials?

Participants were taught using a dartboard example on a whiteboard. The experimenter expressed how one can get better at darts by changing where you aim on the dartboard, or by "subconsciously" improving. We told participants that we were interested in this "subconscious" component of learning. Participants were told to always aim to the target center on every trial. We have update our text to more precisely describe this process:

Lines 534-542: **In contrast to Experiment 1, Participants were instructed to "always aim to the target center and ignore the cursor feedback" for all trials. Participants were also educated on explicit re-aiming strategies using a dartboard example on a whiteboard. The experimenter communicated that performance at darts can improve by changing your aiming location, or by "subconsciously" improving. The experimenter told participants that were interested in this "subconscious" component of learning and therefore to always aim for the target center. Participants repeated the instructions back to the experimenter to confirm understanding.**

Line 507: Is the last trial of every block trial (t-1) or trial (t)?

The last trial of every block is the [t-1] trial. We have labeled trial [t] as the error correction to emphasize how the reinforcement manipulations lead into our main outcome measure, error corrections. We have update our methods to clarify what trial is the last trial of every pseudorandom block:

Line 582: **The last trial [t-1] of every block was an error clamp (Fig. 1C).**

606-610: **After an error clamp trial, participants corrected for the error clamp on the next trial [t]. Trial [t] was the first trial of the next block.**

Line 547: additional word 'in'; "... twice within a block ..."

Thank you. We have fixed this typographical error.
