# Supplementary material for: Successful reinforcement history suppresses explicit and implicit error corrections
Source: PLoS Comput Biol. 2026 Aug 3;22(8):e1014574. doi: 10.1371/journal.pcbi.1014574 (PMC13432137; doi:10.1371/journal.pcbi.1014574)
Supplement: Peer review history

# Peer review: Decision Letter 0

**DOI:** 10.1371/journal.pcbi.1014574.r001

**Authors:** Daniele Marinazzo, Bastien Blain

29 Apr 2026

PCOMPBIOL-D-26-00472

Successful Reinforcement History Suppresses Explicit and Implicit Error Corrections

PLOS Computational Biology

Dear Dr. Buggeln,

Thank you for submitting your manuscript to PLOS Computational Biology. After careful consideration, we feel that it has merit but does not fully meet PLOS Computational Biology's publication criteria as it currently stands. Therefore, we invite you to submit a revised version of the manuscript that addresses the points raised during the review process.

Please submit your revised manuscript by Jun 29 2026 11:59PM. If you will need more time than this to complete your revisions, please reply to this message or contact the journal office at ploscompbiol@plos.org. When you're ready to submit your revision, log on to https://www.editorialmanager.com/pcompbiol/ and select the 'Submissions Needing Revision' folder to locate your manuscript file.

Please include the following items when submitting your revised manuscript:

* A letter that responds to each point raised by the editor and reviewer(s). You should upload this letter as a separate file labeled 'Response to Reviewers'. This file does not need to include responses to formatting updates and technical items listed in the 'Journal Requirements' section below.

* A marked-up copy of your manuscript that highlights changes made to the original version. You should upload this as a separate file labeled 'Revised Manuscript with Track Changes'.

* An unmarked version of your revised paper without tracked changes. You should upload this as a separate file labeled 'Manuscript'.

If you would like to make changes to your financial disclosure, competing interests statement, or data availability statement, please make these updates within the submission form at the time of resubmission. Guidelines for resubmitting your figure files are available below the reviewer comments at the end of this letter

We look forward to receiving your revised manuscript.

Kind regards,

Bastien Blain

Academic Editor

PLOS Computational Biology

Daniele Marinazzo

Section Editor

PLOS Computational Biology

**Journal Requirements:**

If the reviewer comments include a recommendation to cite specific previously published works, please review and evaluate these publications to determine whether they are relevant and should be cited. There is no requirement to cite these works unless the editor has indicated otherwise.

1) Please ensure that the CRediT author contributions listed for every co-author are completed accurately and in full.

At this stage, the following Authors/Authors require contributions: John Buggeln, Jan A. Calalo, Michael J. Carter, Joshua G.A. Cashaback, Nicholas Muscara, Truc T. Ngo, Adam M Roth, Matthew Short, and Seth R. Sullivan. Please ensure that the full contributions of each author are acknowledged in the "Add/Edit/Remove Authors" section of our submission form.

The list of CRediT author contributions may be found here: https://journals.plos.org/ploscompbiol/s/authorship#loc-author-contributions

2) We ask that a manuscript source file is provided at Revision. Please upload your manuscript file as a .doc, .docx, .rtf or .tex. If you are providing a .tex file, please upload it under the item type u2018LaTeX Source Fileu2019 and leave your .pdf version as the item type u2018Manuscriptu2019.

3) Thank you for including an Ethics Statement for your study. Please include:

i) The full name(s) of the Institutional Review Board(s) or Ethics Committee(s)

ii) The approval number(s), or a statement that approval was granted by the named board(s).

4) Please upload all main figures as separate Figure files in .tif or .eps format. For more information about how to convert and format your figure files please see our guidelines:

https://journals.plos.org/ploscompbiol/s/figures

5) We notice that your supplementary Figures are included in the manuscript file. Please remove them and upload them with the file type 'Supporting Information'. Please ensure that each Supporting Information file has a legend listed in the manuscript after the references list.

6) Please amend your detailed Financial Disclosure statement. This is published with the article. It must therefore be completed in full sentences and contain the exact wording you wish to be published.

1) State the initials, alongside each funding source, of each author to receive each grant. For example: "This work was supported by the National Institutes of Health (####### to AM; ###### to CJ) and the National Science Foundation (###### to AM)."

2) State what role the funders took in the study. If the funders had no role in your study, please state: "The funders had no role in study design, data collection and analysis, decision to publish, or preparation of the manuscript."

3) If any authors received a salary from any of your funders, please state which authors and which funders..

If you did not receive any funding for this study, please simply state: u201cThe authors received no specific funding for this work.u201d

6) Some material included in your submission may be copyrighted. According to PLOS's copyright policy, authors who use figures or other material (e.g., graphics, clipart, maps) from another author or copyright holder must demonstrate or obtain permission to publish this material under the Creative Commons Attribution 4.0 International (CC BY 4.0) License used by PLOS journals. Please closely review the details of PLOS's copyright requirements here: PLOS Licenses and Copyright. If you need to request permissions from a copyright holder, you may use the PLOS Content Copyright Permission Form Please respond directly to this email and provide any known details concerning your material's license terms and permissions required for reuse, even if you have not yet obtained copyright permissions or are unsure of your material's copyright compatibility. Once you have responded and addressed all other outstanding technical requirements, you may resubmit your manuscript within Editorial Manager. Potential Copyright Issues:

i) 1, 3, and 4.: Please confirm whether you drew the images / clip-art within the figure panels by hand. If you did not draw the images, please provide (a) a link to the source of the images or icons and their license / terms of use; or (b) written permission from the copyright holder to publish the images or icons under our CC BY 4.0 license. Alternatively, you may replace the images with open source alternatives. See these open source resources you may use to replace images / clip-art: - https://commons.wikimedia.org - https://openclipart.org/

7) Please send a completed 'Competing Interests' statement, including any COIs declared by your co-authors. If you have no competing interests to declare, please state "The authors have declared that no competing interests exist". Otherwise please declare all competing interests beginning with the statement "I have read the journal's policy and the authors of this manuscript have the following competing interests"

**Reviewers' comments:**

Reviewer's Responses to Questions

**Comments to the Authors:**

**Please note here if the review is uploaded as an attachment.**

Reviewer #1: The authors present a study examining the influence of binary reinforcement feedback on explicit and implicit motor corrections made in response to clamped vector error feedback. Three behavioral experiments build on each other to show, collectively, that longer-term reinforcement history seems to preferentially impact explicit error corrections (experiments 1 and 2), while the immediate effect of reinforcement feedback signaling the absence of task error, despite visual feedback of a motor error, can reduce implicit corrections (experiment 3). In addition to behavioral results, the authors include an analysis using models of hypothesized potential mechanisms underlying the observed behavior. This model analysis shows that two candidate models can approximate the observed behavior. Specifically, a model that accounts for the expected value of actions can account for the modulation of explicit error corrections, while an error-based learning model that accounts for the presence of a task error can account for the modulation of implicit corrections. In both cases, the successful models outperformed alternative models/hypothesized mechanisms.

Overall, I found the paper interesting and think it adds to the body of literature attempting to understand the interaction(s) between reinforcement and supervised learning systems. Despite these strengths, I have some concerns/questions that I would like to see addressed before the manuscript is published. My specific comments are below.

1. The mechanistic models and interpretation of the behavioral results in Experiments 1 and 2 make assumptions about the expected value of actions – i.e., that the actions of the 80% group would have a higher expected value than those of the 20% group. However, expected values are typically learned over time, and the analysis of error clamp trials appears to collapse all trials into a single subject mean. If I am correct, participants were not exposed to the probabilistic reinforcement during baseline, so it is not clear that participants would have formed an estimate of expected value until later in the task. Can the authors confirm whether performance was stable over time in their task, and, if so, discuss how this reconciles with typical theories of expected value?

2. Relatedly, did all participants reliably reach within the reward region in Experiments 1 and 2? For example, were there any participants in the 80% group who missed the reward region on enough trials to have had an impact on the total reinforcement rate?

3. For all experiments, the authors report corrections in a single direction (i.e., more positive is larger), but the methods state that the direction of the error clamp was counterbalanced across trials. Am I correct in assuming the authors flipped one of the error-clamp conditions? Given that they are measuring the response to vector error clamp feedback, it is important to ensure that corrections are made in the direction countering the imposed errors; otherwise, it is difficult to be sure that participants were responding to the error at all. Could the authors either clarify how their error corrections are reported or include an analysis of corrections relative to the error direction?

4. In Experiments 1 and 2, why was the baseline variability, which was used to set the width of the reward region, calculated using a mix of trials with and without visual feedback (20 and 20, if I am interpreting the methods correctly)?

5. Were the short history of reward clamp conditions in Experiments 1 and 2 counterbalanced across trials within each reinforcement group? Or did each group get a fixed short-history condition?

6. For Experiment 3, can the authors explain the choice to provide reinforcement feedback whenever participants hit the target in the larger+reinforcement condition, but only provide punishment when participants were in the reward region in the small+punishment condition? How frequently did participants miss the target by reaching outside the reward region in the small+punishment condition? I am concerned that inconsistent punishment feedback could have led participants to discount or ignore it, which could explain the lack of modulation observed in that condition (rather than it resulting from differential processing of reward versus punishment).

7. It is not totally clear how the authors think the results and top-performing models from the three experiments presented here go together. In Experiments 1 and 2, the short-term history of reward is nested within the long-term history. That is, to have a reward prediction error, the system must have some knowledge of the reward history. In Experiment 3, however, the immediate history of reward is treated as something independent of any representation of the reward history. Could the authors clarify their thinking about this in the discussion, or when justifying the design of Experiment 3?

8. The authors’ interpretation of their results seems to treat explicit and implicit error corrections as non-overlapping, but it is likely that both correction processes operate together to drive behavior in daily life. I am wondering whether the authors could discuss the ways their results elucidate the interaction between explicit and implicit corrections. I think this issue is particularly relevant when interpreting the result of Experiment 3, which included a manipulation of task errors. The authors point out that task errors are considered by some to be reinforcement signals, but task errors are also thought to be a significant driver of explicit corrections (and that the link to reinforcement learning is via explicit mechanisms).

9. I noticed a few typographical errors:

Page 2, line 55, should be modulates

Page 14, line 242, either “corrected” or “to” is incorrect here

Page 26, line 531, missing “subjects”

Reviewer #2: In the article “Successful reinforcement history suppresses explicit and implicit error corrections,” the authors look to determine the impact of reinforcement history on explicit (cognitive) and implicit (involuntary) motor corrections. Three experiments were conducted in which the authors compared two competing hypotheses: (1) reward prediction errors (surprise) modulate explicit and implicit error corrections and (2) expected value (average reward) modulates explicit and implicit error corrections. Results, experimental behaviour and computational modeling, suggest that expected value modulates explicit error corrections. An immediate history of reinforcement modulates implicit error corrections. Together, results indicate that a successful reinforcement history suppress explicit and implicit error corrections.

The authors ask an interesting question, and I appreciate the clever methodology adapted to manipulate reward prediction errors and expected value. Below I outline several questions, mainly dealing with clarification around the methodology.

The word reinforcement is used to refer to movement outcome (success or failure), as well as additional extrinsic feedback. How do these reinforcement sources interact?

To clarify methodology:

1. Do participants see cursor feedback throughout the reach? If not, how does this influence sensory prediction errors and underlying implicit corrections?

2. Participants were to move through the target. If they reached through a reward zone, they then had a 20% or 80% chance of receiving feedback on each trial in Experiments 1 and 2. Did this ensure that participants received feedback on 2 and 8 of the 10 initial trials?

3. In the error clamp trials (t-3 and t-2), did the cursor feedback go through the target? What happened on trial (t-2) if reinforcement feedback was not provided?

4. In Figure caption 1 it indicates that reinforcement feedback was not contingent on performance. Is this the case? Previous text indicated that feedback was only provided if reaches were through a reward region.

5. Can the reinforcement and punishment feedback be considered equivalent? Is the reinforcement feedback processed as rewarding as the punishment feedback is processed as punishing?

6. Was the target shown before the go signal? If so, how did this long preparation time influence the engagement of explicit/implicit processes?

7. Was there only 1 target? How does use-dependent error corrections/learning factor into the current results?

Is the factor of the short-history of reinforcement clamps factored into the results (RC1 versus RC2)?

Was the factor of block (i.e., time) considered in the current results? Previous research has shown early adjustments in reaches are typically driven by explicit processes, with implicit contributions increasing over trials.

Conclusions drawn (e.g., lines 204-206; 289-291), assume that explicit and implicit corrections are independent. Research has shown that there are multiple explicit and implicit processes, and that these processes may interact (see Weightman et al., 2022; Larssen and Hodges, 2023).

Minor

Line 2: Does a basketball player expect to make many shots and is a miss surprising? Average shooting percentage, even for professionals, is less than 50%, so technically players miss more shots than they make.

Line 64: missing a word; “ …, but could have been through …”

Line 74: please clarify reward valence.

Figure caption 1: missing the word ‘to’; “from the 13th to 4th trial prior to an error correction …”

Figure caption 1: the phrase ‘participants were shown an error clamp (t-1) is unclear. Did participants not experience an error clamp trial as they reached?

Line 210: the phrase “immediate history that could be provided on the same trial as the error clamp” is confusing. Are you referring to the extrinsic reinforcement/punishment feedback being provided on trial (t-1) with the error clamp?

Line 318: missing the word ‘a’; “… but they did find a difference …”

Line 321: How gradients of feedback?

Line 478: How were participants educated on explicit re-aiming strategies? What were they told re the error clamp trials?

Line 507: Is the last trial of every block trial (t-1) or trial (t)?

Line 547: additional word ‘in’; “… twice within a block …”

**********

**Have the authors made all data and (if applicable) computational code underlying the findings in their manuscript fully available?**

The PLOS Data policy requires authors to make all data and code underlying the findings described in their manuscript fully available without restriction, with rare exception (please refer to the Data Availability Statement in the manuscript PDF file). The data and code should be provided as part of the manuscript or its supporting information, or deposited to a public repository. For example, in addition to summary statistics, the data points behind means, medians and variance measures should be available. If there are restrictions on publicly sharing data or code —e.g. participant privacy or use of data from a third party—those must be specified.

Reviewer #1: Yes

Reviewer #2: None

**********

PLOS authors have the option to publish the peer review history of their article (what does this mean?). If published, this will include your full peer review and any attached files.

If you choose “no”, your identity will remain anonymous but your review may still be made public.

**Do you want your identity to be public for this peer review?**  For information about this choice, including consent withdrawal, please see our Privacy Policy.

Reviewer #1: No

Reviewer #2: No

**Figure resubmission:**

While revising your submission, we strongly recommend that you use PLOS’s NAAS tool (https://ngplosjournals.pagemajik.ai/artanalysis) to test your figure files. NAAS can convert your figure files to the TIFF file type and meet basic requirements (such as print size, resolution), or provide you with a report on issues that do not meet our requirements and that NAAS cannot fix.

After uploading your figures to PLOS’s NAAS tool - https://ngplosjournals.pagemajik.ai/artanalysis, NAAS will process the files provided and display the results in the "Uploaded Files" section of the page as the processing is complete. If the uploaded figures meet our requirements (or NAAS is able to fix the files to meet our requirements), the figure will be marked as "fixed" above. If NAAS is unable to fix the files, a red "failed" label will appear above. When NAAS has confirmed that the figure files meet our requirements, please download the file via the download option, and include these NAAS processed figure files when submitting your revised manuscript.

**Reproducibility:**

To enhance the reproducibility of your results, we recommend that authors of applicable studies deposit laboratory protocols in protocols.io, where a protocol can be assigned its own identifier (DOI) such that it can be cited independently in the future. Additionally, PLOS ONE offers an option to publish peer-reviewed clinical study protocols. Read more information on sharing protocols at https://plos.org/protocols?utm_medium=editorial-email&utm_source=authorletters&utm_campaign=protocols

# Author comment: Author response to Decision Letter 1

**DOI:** 10.1371/journal.pcbi.1014574.r002

19 May 2026

Attachment Submitted filename: ResponsetoReviewers.pdf

# Peer review: Decision Letter 1

**DOI:** 10.1371/journal.pcbi.1014574.r003

**Authors:** Daniele Marinazzo, Bastien Blain

14 Jul 2026

Dear Mr. Buggeln,

We are pleased to inform you that your manuscript 'Successful Reinforcement History Suppresses Explicit and Implicit Error Corrections' has been provisionally accepted for publication in PLOS Computational Biology, provided that you correct and clarify the sentences pointed out by the reviewers.

Before your manuscript can be formally accepted you will need to complete some formatting changes, which you will receive in a follow up email. A member of our team will be in touch with a set of requests.

Please note that your manuscript will not be scheduled for publication until you have made the required changes, so a swift response is appreciated.

IMPORTANT: The editorial review process is now complete. PLOS will only permit corrections to spelling, formatting or significant scientific errors from this point onwards. Requests for major changes, or any which affect the scientific understanding of your work, will cause delays to the publication date of your manuscript.

Should you, your institution's press office or the journal office choose to press release your paper, you will automatically be opted out of early publication. We ask that you notify us now if you or your institution is planning to press release the article. All press must be co-ordinated with PLOS.

Thank you again for supporting Open Access publishing; we are looking forward to publishing your work in PLOS Computational Biology.

Best regards,

Bastien Blain

Academic Editor

PLOS Computational Biology

Daniele Marinazzo

Section Editor

PLOS Computational Biology

***********************************************************

Reviewer's Responses to Questions

**Comments to the Authors:**

**Please note here if the review is uploaded as an attachment.**

Reviewer #1: The authors have satisfactorily revised the manuscript. I have only very minor comments on typographical and grammatical errors that likely resulted from adding text.

Line 155 - missing an "s" on "participant"

Line 200 - "nor" should be "or"

Lines 216-18 - Something is not right about the sentence beginning "Our behavioral..."

Line 236 - Missing a comma

Reviewer #2: This is my second review of the article “Successful reinforcement history suppresses explicit and implicit error corrections.” I appreciate the effort the authors have taken to address the previous round of questions/comments raised by myself and the other reviewer.

Below, I indicate lines of text that could be clarified for the reader.

Lines 25-28: Despite knowledge that key reinforcement computations depend on history,1,2 it is unclear how reinforcement history specifically interacts with error corrections. Understanding these processes’ interactions and temporal dynamics is not just a theoretical exercise, but can generate neurorehabilitation improvements.

It is unclear which processes are being referred to with respect to ‘processes’ interactions’. I am not sure I would categorize reinforcement history as a process.

Lines 39-41: Reinforcement feedback independently regulates movement variability,29–32 recalibrates the sensorimotor system,33 and drives plasticity changes in the motor cortex.

The words plasticity and change are not needed.

Lines 106-108: We experimentally controlled the error size to assess participants’ error corrections [i.e., t] on the next trial.

I would place [i.e., t] at the end of the sentence, as t does not refer to error corrections; “… on the next trial [i.e., t].”

Lines 164-166: We confirmed that participants in the 20% and 80% Reinforcement group had different actual reward rates (18.35% vs. 67.60%, p <0.001).

It would be good to indicate the response rates associated with each group (e.g., by putting in the word ‘respectively’). This is also found at Lines 202-204.

Lines 269-271: Fig. 4B shows a representative participant illustrating how the feedback conditions were pseudorandomized and how participants responded to error clamps.

It would be good to indicate that this figure shows data – even what type of data – for a representative participant.

Lines 316-317: Our experiments and models suggest that a higher expected value decreases explicit error corrections.

This sentence is rather vague. It would be good to indicate what is being referred to with respect to higher expected value.

Lines 516-519: Endpoint feedback has been used effectively to induce implicit and explicit error corrections.7,8,15,81 Endpoint feedback also prevents feedback error corrections, which ensures our metrics were measuring feedforward motor commands.

Given the nature of your task and endpoint feedback, I assume that the explicit and implicit corrections you are referring to are on the next trial (or subsequent trials). It would be good to specify that these are offline corrections. The term ‘online’ could also be used in the second sentence to refer to feedback-based corrections during a current trial.

Lines 588-592: The explicit process can compensate with greater magnitude and more quickly to large errors. Also, implicit corrections peak at error sensitivity at smaller error sizes.85

These lines are difficult to follow. I assume the comparison in the first sentence is to the implicit process. I am not sure what is meant by ‘peak at error sensitivity at smaller error sizes.’

**********

**Have the authors made all data and (if applicable) computational code underlying the findings in their manuscript fully available?**

The PLOS Data policy requires authors to make all data and code underlying the findings described in their manuscript fully available without restriction, with rare exception (please refer to the Data Availability Statement in the manuscript PDF file). The data and code should be provided as part of the manuscript or its supporting information, or deposited to a public repository. For example, in addition to summary statistics, the data points behind means, medians and variance measures should be available. If there are restrictions on publicly sharing data or code —e.g. participant privacy or use of data from a third party—those must be specified.

Reviewer #1: Yes

Reviewer #2: Yes

**********

PLOS authors have the option to publish the peer review history of their article (what does this mean?). If published, this will include your full peer review and any attached files.

If you choose “no”, your identity will remain anonymous but your review may still be made public.

**Do you want your identity to be public for this peer review?**  For information about this choice, including consent withdrawal, please see our Privacy Policy.

Reviewer #1: No

Reviewer #2: No

# Editor report: Acceptance letter

**DOI:** 10.1371/journal.pcbi.1014574.r004

**Authors:** Daniele Marinazzo, Bastien Blain

PCOMPBIOL-D-26-00472R1

Successful Reinforcement History Suppresses Explicit and Implicit Error Corrections

Dear Dr Buggeln,

I am pleased to inform you that your manuscript has been formally accepted for publication in PLOS Computational Biology. Your manuscript is now with our production department and you will be notified of the publication date in due course.

The corresponding author will soon be receiving a typeset proof for review, to ensure errors have not been introduced during production. Please review the PDF proof of your manuscript carefully, as this is the last chance to correct any errors. Please note that major changes, or those which affect the scientific understanding of the work, will likely cause delays to the publication date of your manuscript.

Soon after your final files are uploaded, unless you have opted out, the early version of your manuscript will be published online. The date of the early version will be your article's publication date. The final article will be published to the same URL, and all versions of the paper will be accessible to readers.

For Research, Software, and Methods articles, you will receive an invoice from PLOS for your publication fee after your manuscript has reached the completed accept phase. If you receive an email requesting payment before acceptance or for any other service, this may be a phishing scheme. Learn how to identify phishing emails and protect your accounts at https://explore.plos.org/phishing.

Thank you again for supporting PLOS Computational Biology and open-access publishing. We are looking forward to publishing your work!

With kind regards,

Anitha Samidurai

PLOS Computational Biology | Carlyle House, Carlyle Road, Cambridge CB4 3DN | United Kingdom ploscompbiol@plos.org | Phone +44 (0) 1223-442824 | ploscompbiol.org | @PLOSCompBiol
